# Toxic Concentrations of Glutamate Induce Ferroptosis without Affecting Mitochondrial Respiration in Rodent Brain Slice Cultures

**DOI:** 10.1523/ENEURO.0439-25.2026

**Published:** 2026-10-29

**Authors:** Annette Vaglio-Garro, Marco Theuermann, Katharina E. Sweis, Sergejs Zavadskis, Márton Kokas, Zsuzsanna Környei, Miriam Karas, Laszlo Tretter, Tímea Komlódi, Adelheid Weidinger, Andrey V. Kozlov

**Affiliations:** ^1^Ludwig Boltzmann Institute for Traumatology, The Research Centre in Cooperation with AUVA, Vienna 1200, Austria; ^2^Austrian Cluster for Tissue Regeneration, Vienna 1200, Austria; ^3^Centro de Investigación en Enfermedades Tropicales (CIET), Universidad de Costa Rica, San José 2060, Costa Rica; ^4^Instituto de Investigaciones en Salud (INISA), Universidad de Costa Rica, San José 2060, Costa Rica; ^5^Department of Biochemistry, Semmelweis University, Budapest 1094, Hungary; ^6^Momentum Laboratory of Neuroimmunology, HUN-REN Institute of Experimental Medicine, Budapest 1083, Hungary

**Keywords:** cell death, excitotoxicity, ferroptosis, glutamate

## Abstract

Many neuronal disorders are characterized by glutamate toxicity, which is closely associated with mitochondrial and neuronal dysfunction. The aim of this study was to investigate the link between mitochondrial function and mechanisms of cell death induced by glutamate. The study was carried out on male (C57BL/6) mice and male Sprague Dawley rat acute brain slices (ABSs), as well as male and female organotypic brain slice cultures (BSCs). Mitochondrial respiration, activities of tricarboxylic acid cycle enzymes, and the cell death rate [assessed by lactate dehydrogenase release and histological analyses] were determined. Memantine was used to inhibit excitotoxicity, while ferrostatin-1, rosiglitazone, and deferoxamine were used as inhibitors of the ferroptotic pathway. In astrocytes, pyruvate dehydrogenase complex and oxoglutarate dehydrogenase complex (OGDHC) showed <1% of the enzymatic activity observed in the whole-brain tissue. In BSCs, glutamate exposure induced a marked increase in oxidative phosphorylation-linked mitochondrial respiration, which was independent of OGDHC activity and disappeared when BSC homogenates were analyzed. Glutamate-induced cell death was not alleviated by memantine but was significantly reduced by ferrostatin-1. The activation of ferroptosis by glutamate was also observed in ABSs, as well as the inhibition of lipid peroxidation induced by ferrostatin-1, rosiglitazone, and deferoxamine. Ferrostatin-1 and rosiglitazone additionally inhibited cell death, while deferoxamine did not. The unusual response to deferoxamine requires further elucidation. In summary, our data suggest that elevated glutamate levels enhance neuronal energy metabolism in brain slices, compensating glutamate-mediated Ca^2+^ influx and reduce excitotoxicity. In contrast, persistently elevated levels of extracellular glutamate impair the cystine/glutamate antiporter, leading to ferroptosis.

## Graphical abstract

**Figure:**
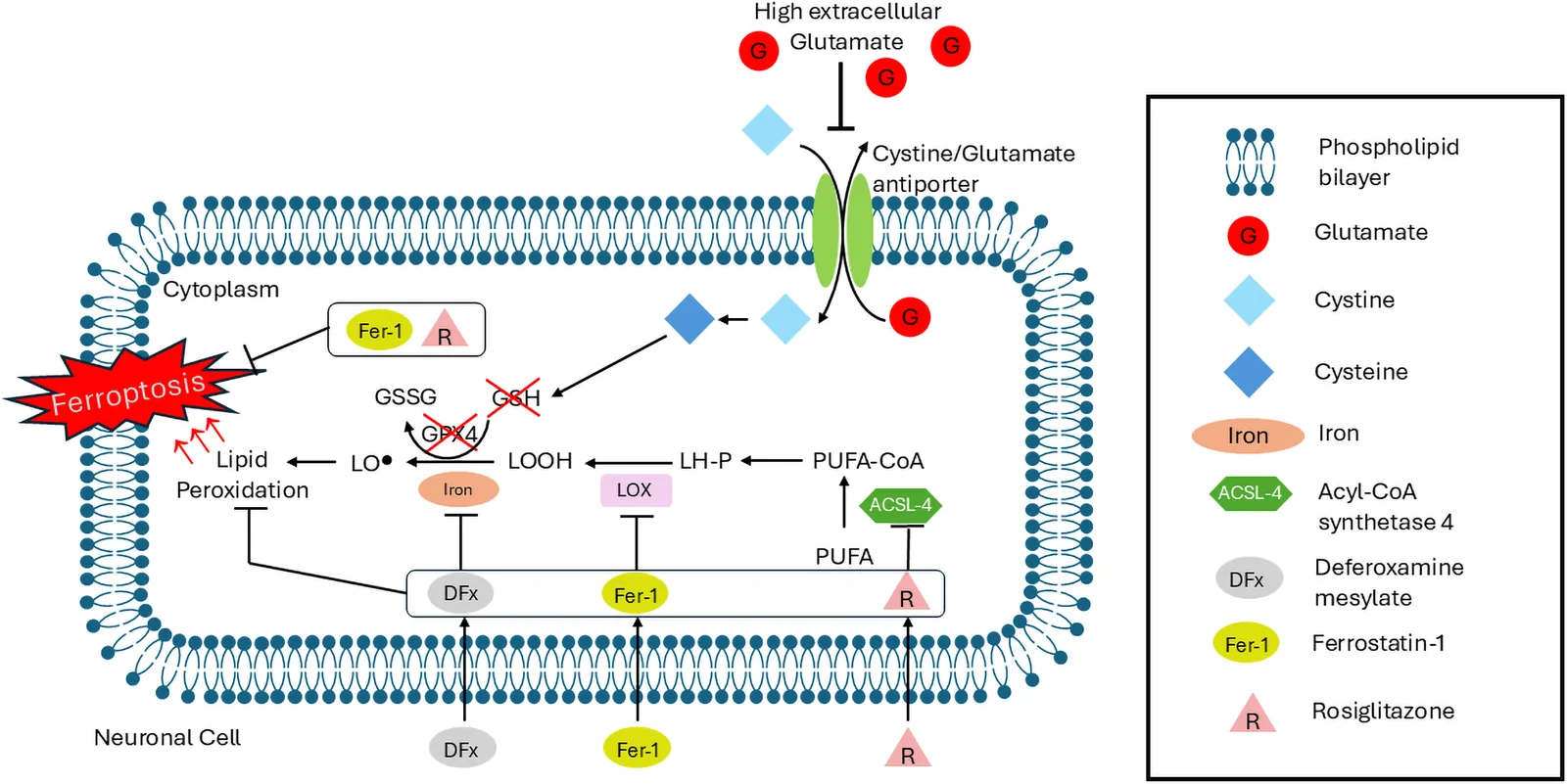
Visual Abstract

## Significance Statement

Excessive extracellular glutamate is a hallmark of many neurodegenerative and ischemic conditions. This study reveals that toxic glutamate concentrations in brain slice cultures, modeling brain tissue, intensify neuronal energy metabolism without rescuing cells from death, shifting the predominant mechanism from excitotoxicity to ferroptosis. Such phenomenon was not observed in studies on neuronal cell cultures, also including our previous studies. These results suggest that targeting ferroptosis may offer therapeutic potential in neuronal disorders traditionally attributed to excitotoxic damage.

## Introduction

Brain injury (Ray et al., 2002) or neurodegenerative pathologies (Doble, 1999) can induce accumulation of extracellular glutamate in the brain to toxic concentrations. These toxic levels of glutamate induce morphological changes in neurons, manifesting in deformed dendrites and expanded rounded neurons (Reynolds’ and Hastings, 1995; Jellinger, 1999; Adamchik et al., 2000; Robert et al., 2002; Youdim et al., 2002; García et al., 2005), showing degenerative cell (Kang et al., 2020; Erkartal et al., 2025) and neuronal death (Weidinger et al., 2023). In primary neuronal cultures, it has been shown that glutamate toxicity primarily targets neurons, as evidenced by a marked reduction in the number of tyrosine hydroxylase-positive neurons together with neurite degeneration (Weidinger et al., 2023). Glutamate-induced cell death is associated with mitochondrial dysfunction, associated with accumulation of Ca^2+^, activating mitochondrial permeability transition pore opening and causing functional collapse of mitochondria (for review, see Szabó and Zoratti, 1992; Bonora et al., 2020). In addition, Ca^2+^ stimulates the induction of apoptosis via release of cytochrome c (cyt c) and apoptosis inducing factor (Lemasters et al., 2002).

In a previous study, restoration of the mitochondrial enzyme complex oxoglutarate dehydrogenase complex (OGDHC) activity attenuated glutamate-induced Ca^2+^ accumulation and neuronal cell death (Weidinger et al., 2023), supporting a mechanistic link between glutamate metabolism, excitotoxic Ca^2+^ signaling, and neuronal survival.

Recently iron-mediated lipid peroxidation (LPO) reactions have been recognized as the major contributor to a novel mechanism of programmed cell death known as ferroptosis (Stoyanovsky et al., 2019), which has been also associated with neural disorders (Li et al., 2019; Wang, T. et al., 2022; Wang, Z. L. et al., 2022).

Under normal circumstances, glutathione peroxidase (GPX) neutralizes lipid peroxide to lipid alcohols, but the depletion of intracellular glutathione (GSH, GPX substrate) reduces GPX functionality (Yang and Stockwell, 2008; Friedmann Angeli et al., 2014). Glutathione required for the GPX activity is synthesized from cystine, which is transported into the cell via cystine/glutamate (Xc^−^) antiporter. Since elevated extracellular glutamate concentration blocks this transporter, it may induce ferroptosis.

Under physiological conditions, the extracellular glutamate enters the glutamine–glutamate cycle (McKenna et al., 2016; Sonnewald and Schousboe, 2016). A part of the glutamate can be taken up by the mitochondria, where it is metabolized to 2-oxoglutarate further contributing to the synthesis of ATP (Tang and Zucker, 1997; Verstreken et al., 2005), thereby removing it from the glutamine–glutamate cycle and reducing its toxicity (Vaglio-Garro et al., 2024b).

Neuronal energy metabolism is predominantly based on the oxidation of glucose. Glutamate is reserved for signal transduction and is normally not used for energy purposes; however, if pyruvate is not sufficiently present in the mitochondria, then glutamate can be used instead (Sonnay et al., 2017). Glutamate dehydrogenase (GDH) and OGDHC (also known as alpha-ketoglutarate dehydrogenase complex) are responsible for up taking glutamate into the tricarboxylic acid (TCA) cycle. This pathway mainly occurs in neurons (Dobolyi et al., 2020; Zhong et al., 2022), and OGDHC is a limiting step in this process (Mkrtchyan et al., 2018). In contrast to neurons, astrocytes obtain their energy predominantly from glycolysis (Brooks, 2018).

It has been shown that memantine, an *N*-methyl-d-aspartate (NMDA) receptor antagonist, protected neuronal primary cells from glutamate-induced death (Weidinger et al., 2023). However, it is still not completely clear whether this mechanism operates in the brain tissue as well.

Brain slice culture (BSC) is an in vitro model in the field of neurosciences that represents the architecture of the brain (Beach et al., 1982; Bahr, 1995; Sieg et al., 1998). Advantages of this model are: plasticity (Simoni et al., 2003), three-dimensional structure and organization (Bahr, 1995), the existence of network connections (Ito et al., 2014), and the presence of different populations of brain cells (Beach et al., 1982; Staal et al., 2011; Croft et al., 2019) even when they have kept in culture for weeks to months preserving their 3D architecture, neuronal networks, and cell environment (Gähwiler et al., 1997). BSC provides more complex and physiologically relevant model compared with traditional dissociated neuronal cultures (Humpel, 2018).

Because they preserve their anatomically brain regions, it is also possible to recapitulate pathological changes in energy metabolism because the slices are highly sensitive to oxygen and glucose changes (Underwood et al., 2020).

The disadvantage of this model is that at this stage the brain does not reach the adult phase of development. The acute brain slices (ABSs) have been developed and used for investigating the structural and functional characteristics of the adult brain (Eguchi et al., 2020). In this study, we investigated both BSCs and ABSs to access the mechanisms underlying glutamate-mediated cell death.

The aim of this study was to investigate mitochondrial function and cell death pathways in BSCs and ABSs that are exposed to toxic concentration of glutamate and compare these results with those obtained in cell cultures.

## Materials and Methods

### Institutional review Board Statement

The animal study protocol was approved by the Institutional Review Board (Tierschutzgremium) of the Ludwig Boltzmann Institute of Traumatology (Certification MA58-821/90) on February 16, 2023.

#### Housing of mice

Male (C57BL/6) mice were housed at the minimal disease level under standard environmental conditions: 20–22°C, 12 h light/dark cycles, and 65% humidity. Animals were fed with standard pellet chow [55% carbohydrate, 27% protein, 7% lipid (Garant)] optimal for rodents. Food and water were available *ad libitum*. Newborn mice were fed by their mothers. Experiments were compiled in accordance with the International Guiding Principles for Biomedical Research Involving Animals and Guidelines for Animal Experiments at Semmelweis University according to the EU Directive “Directive 2010/63/EU of the European Parliament and of the Council of 22 September 2010 on the protection of animals used for scientific purposes.”

#### Isolation of astrocytes

Primary astrocytes were isolated from the forebrains of 1- or 2-d-old newborn mice (Környei et al., 2005; 2007). Following decapitation, the skin and skull were carefully removed, and the brains were transferred to Petri dishes containing phosphate-buffered saline (PBS; Sigma-Aldrich) supplemented with 5 µg/ml amphotericin B. The meninges were gently peeled away using fine forceps under a stereomicroscope, and the brains were subsequently minced into small pieces with a sterile blade. The tissue pieces were subjected to enzymatic dissociation using 0.05% w/v trypsin (Sigma-Aldrich) for 10 min. Following homogenization and subsequent centrifugation (6 min, 220 × *g*), the cells were plated onto poly-ornithine-coated (Sigma-Aldrich) Petri dishes in minimum essential medium (MEM; Sigma-Aldrich, M2279) supplemented with 10% heat-inactivated fetal bovine serum (Thermo Fisher Scientific–Invitrogen), 4 mM l-glutamine (Sigma-Aldrich), 2.5 µg/ml amphotericin B (Sigma-Aldrich), and 0.04 mg/ml gentamicin (Sandoz). The cultures were maintained in humidified air atmosphere containing 5% CO_2_ at 37°C. The culture medium was changed on the first 2 d and every second or third day afterward. The astroglia cultures reached 90% confluency within a week. To limit undue expansion of microglia, cultures were maintained for no longer than 13 d. Cell counting was performed using Trypan blue exclusion.

#### Isolation of brain mitochondria

Mitochondria from the mouse brain were isolated as described previously (Komlódi et al., 2018; Sváb et al., 2021). In brief, mice were decapitated, and then the brain was removed delicately from the skull and placed into ice-cold Buffer A, pH 7.4 (in mM: 225 mannitol, 75 sucrose, 5 HEPES, 1 EGTA; Sigma-Aldrich). The brain was cut into small pieces and homogenized using a glass/Teflon Potter–Elvehjem homogenizer. The homogenate was centrifuged two times; then it was suspended in 15% Percoll solution (Sigma-Aldrich) and layered on the top of a 23% and a 40% Percoll layer. Thereafter, mitochondrion fraction was centrifuged two more times in ice-cold Buffer A; then pellet was resuspended in Buffer B, pH 7.4 (equal with Buffer A but without EGTA) yielding ∼30 mg/ml protein concentration. Mitochondria were used within 4 h.

#### Determination of enzymatic activities

All enzyme activity experiment were carried out in 50 mM KH_2_PO_4_, pH 7.4, buffer (Sigma-Aldrich) using JASCO V-650 spectrophotometer (ABL&E-JASCO). Total volume of the medium was 2 ml, containing either 1*10^6^ astrocytes (previously determined by trypan blue staining) or 0.1 mg brain mitochondria [previously determined by modified Biuret reaction (Gornall, Bardawill, and David, 1949; Kokas et al., 2025)]. Membranes were broken by 0.1% v/v Triton X-100 (Sigma-Aldrich). NAD(P)H production or consumption was followed at 340 nm, and enzyme activity was calculated with the help of theoretical molar extinction coefficient of the NAD(P)H (*ε* = 6,220 L * mol^−1^ * cm^−1^).

OGDHc (KGDHc) and aconitase activity was measured as described earlier by Kokas et al. (2025). Pyruvate dehydrogenase complex (PDHC) activity was detected as characterized by Elnageh and Gaitonde (1988) with few modifications. Briefly, the medium (which has already contained Triton X-100 and astrocytes or mitochondria) was supplemented with 2 mM EGTA, 1 mM MgCl_2_, 2.5 mM NAD^+^, 0.2 mM TPP, 1 µM rotenone, 6.5 µM PMS, and 0.6 mM INT. The reaction started with the addition of 5 mM pyruvate and 0.2 mM CoA-SH. GDH was measured as described earlier by our group (Elnageh and Gaitonde, 1988) with minor modifications. Shortly, the medium (which has already contained Triton X-100 and astrocytes or mitochondria) was supplemented with 2 mM EGTA, 1 mM MgCl_2_, 1 µM rotenone, and 0.25 mM NADH. The reaction was started by the addition of 5 mM α-KG and 0.12 mM CoA-SH. MDH activity was determined as earlier reported by Tretter and Ádám-Vizi (Tretter and Adam-Vizi, 2000) with minor adjustments. Shortly, the medium (which has already contained Triton X-100 and astrocytes or mitochondria) was supplemented with 2 mM EGTA, 1 mM MgCl_2_, 1 µM rotenone, and 0.25 mM NADH. The reaction was started by the addition of 0.4 mM oxaloacetate. All chemicals and reagents were obtained from Sigma-Aldrich.

#### Isolation of brains

Janvier Labs provided us with pregnant Sprague Dawley rats. Complete brains were isolated from male and female 6 d postnatal Sprague Dawley rats. Additional experiments were conducted in adult male rats (300–350 g) to confirm that the observed effects are also present in the mature brain tissue. The euthanasia by decapitation was done following the rules of the Austrian animal and experimental protection law and European regulations (TVA Number, GZ: 2155234_2022). Brain isolation was done following the protocol from Hotka et al. (2020). Briefly, brains were collected in an ice-cold buffer containing 137 mM NaCl (Sigma-Aldrich), 5.4 mM KCl (Sigma-Aldrich), 1.1 mM Na_2_HPO_4_ (Sigma-Aldrich), 1.1 mM KH_2_PO_4_ (Sigma-Aldrich), 6.1 mM glucose (Sigma-Aldrich), and 1 mM kynurenic acid (Sigma-Aldrich), with pH adjusted to 7.3 using NaOH (Sigma-Aldrich). The whole brain was extracted; meninges, ocular bulbs, cerebellum, pons, pyramidal tract, and medulla were removed. The remaining part of the brain was used for slicing.

#### Preparation of membrane inserts

Six-well plates (Corning Costar) were filled with 1 ml prewarmed (37°C) cell culture medium [50% MEM + HEPES (Thermo Fisher Scientific) supplemented with 25% heat-inactivated horse serum (Thermo Fisher Scientific), 25% Hank´s solution (Thermo Fisher Scientific), 6.5 mg/ml glucose (Fluka BioChemika), and 1% penicillin/streptomycin (Thermo Fisher Scientific)], pH 7.2, and one 30 mm millicell membrane (Millipore, Merck) was inserted into each well.

#### Postnatal organotypic BSCs

Brain slices were prepared following the protocol by Stoppini et al. (1991) and Humpel (2018; Stoppini et al., 1991; Humpel, 2018) using a microtome (Compresstome VF-300, Precisionary Instruments; Weidinger et al., 2013). Briefly, the microtome was disinfected with 70% ethanol and irradiated 30 min with UV light before use. The buffer tank of the microtome was filled with ice-cold Custodiol solution (Dr. F. Köhler Chemie). One side hemisphere was attached with cyanoacrylate-based superglue to the tissue holder in the sagittal direction and fixed with agarose 2% (Sigma-Aldrich). Each hemisphere was sliced in sagittal direction with a microtome in 494 µm thickness with maximum potential of blade force. Slices were grabbed with round tip curved tweezers from the agarose side and placed on top 30 mm millicell membrane (Millipore, Merck) previously prepared. Slices were cultured in humidified atmosphere at 37°C and 5% CO_2_. After 2 d of incubation, the cell culture medium was changed to 50% MEM + HEPES no glutamine (Thermo Fisher Scientific) supplemented with 25% horse serum heat inactivated (Thermo Fisher Scientific), 25% Hank's solution (Thermo Fisher Scientific) and 6.5 mg/ml of glucose (Fluka BioChemika), pH 7.2, medium. The medium was regularly changed two times per week (Mondays and Fridays). Slices were incubated and treated after 14 d of culture. Extended Data Figure 1-1 shows the timeline of the experiment and experimental conditions. Slices coming from the same brain had considered one biologically replicate. For the Seahorse experiments, slices from three to four brains were pooled each time and randomly assigned to different treatments. At least three slices were included per experiment. Each slice, regardless of which pup it was obtained from, was treated as an independent sample. The number of samples is shown in the graphs.

#### Pretreatment with inhibitors and treatment with glutamate of the postnatal BSC

Inhibitor media were prepared by diluting concentrated stock solutions in serum-free cell culture medium [75% MEM + HEPES, glutamine free (Thermo Fisher Scientific)] and 25% Hank's solution (Thermo Fisher Scientific) supplemented with 6.5 mg/ml of glucose (Fluka BioChemika), pH 7.2 (Zou and Crews, 2005; Dixon et al., 2012) to a final concentration of 10 µM ferrostatin-1 [Eurobio Scientific (Li et al., 2017)] or 120 µM memantine [Sigma-Aldrich (Hou et al., 2014; Ma et al., 2023; Carrillo et al., 2024)] or 50 µM AP-5 [Sigma-Aldrich (Sebastião et al., 2001; Andrew et al., 2022)] + 20 µM CNQX [Sigma-Aldrich (Sebastião et al., 2001; Andrew et al., 2022)] and 30 mM glutamate [Sigma-Aldrich (Ferreira et al., 2021)]. On Day 15, 30 mm millicell membranes (Millipore, Merck) containing slices were transferred to fresh six-well plates containing 1 ml of inhibitor medium per well. Slices were incubated with media containing inhibitors for 30 min at 37°C in a humidified atmosphere containing 5% CO_2_. After the inhibitor media were removed, 1 ml of glutamate dilution was added per well; the 30 mm millicell membrane (Millipore, Merck) with slices was inserted into the wells and incubated for 2 h (Ferreira et al., 2021) at 37°C in a humidified atmosphere containing 5% CO_2_. Millicell membrane with brain slices was moved into fresh 1 ml of 75% MEM + HEPES, glutamine free (Thermo Fisher Scientific) supplemented with 25% Hank's solution (Thermo Fisher Scientific) and 6.5 mg/ml of glucose (Fluka BioChemika) free serum medium, pH 7.2, the samples were incubated at 37°C in a humidified atmosphere containing 5% CO_2_ for 24 h. After the incubations, some slices were homogenated and used to measure mitochondrial respiration in Oroboros Instruments, and some slices were punched and used to measure mitochondrial respiration on Seahorse.

Organotypic brain slices culture is very versatile to develop long-term pharmacological molecules impact, protein function, neuronal physiology, and others. These slices can be kept in culture for weeks to months preserving their 3D architecture, neuronal networks, and cell environment (Gähwiler et al., 1997). Because they preserve their anatomically brain regions, it is possible to develop mitochondrial energetics changes because the slices are highly sensitive to oxygen and glucose changes (Underwood et al., 2020).

#### Postnatal brain slice homogenization

The weight of three brain slices was determined, and they were homogenized in the following buffer: 0.25 M saccharose (Sigma-Aldrich), 10 mM Tris, 0.5 mM EDTA (Sigma-Aldrich), 0.5 mg/ml fatty acid-free bovine serum albumin (Sigma-Aldrich), pH 7.2.

#### Measurement of mitochondrial respiration in postnatal BSC

##### In brain homogenate

Mitochondrial respiratory activity was measured with high-resolution respirometry (Oroboros O2k, Oroboros Instruments), and data were recorded using the DatLab software (Oroboros Instruments). A combined protocol was performed as previously described (Weidinger et al., 2023), in which we measured Complex II after the inhibition of Complex I. To initiate mitochondrial respiration, we provided electrons to the electron transport system (ETS) through Complex I through addition of 10 mM glutamate (Sigma-Aldrich) as a substrate which generated NADH (leak respiration). Next, we stimulated ATP synthesis by adding 1 mM ADP (Sigma-Aldrich) to the chamber. These steps allowed us to estimate the contribution of the Complex I linked respiration. To dissect the contribution of Complex II, 1.4 mM of the Complex I inhibitor rotenone (Sigma-Aldrich) was added. Subsequently, 10 mM of Complex II substrate succinate (Sigma-Aldrich) was introduced, thus now driving the respiration exclusively through Complex II. Next, maximal respiratory capacity of the ETS was measured after stepwise titration with 0.3 µM of the uncoupler carbonyl cyanide 4-(trifluoromethoxy) phenylhydrazone (FCCP; Sigma-Aldrich). To assess mitochondrial outer membrane integrity, 2.5 µM exogenous cyt c (Sigma-Aldrich) was added, with a rise in respiration at this point would indicate loss of endogenous cyt c. Finally, mitochondrial respiration was terminated by the addition of 12.5 µM Complex III inhibitor myxothiazol (Sigma-Aldrich).

##### In tissue

The ATP Rate Assay on the Agilent Seahorse XF24 analyzer was used to assess mitochondrial respiration. Three-millimeter punches of treated 250 µm cortical brain slices were transferred to Islet Capture Microplates, 0.5 ml XF DMEM Medium pH 7.4 (Seahorse, Agilent Technologies). Glucose (10 mM; Fluka BioChemika) and pyruvate (2 mM; Sigma-Aldrich) were added per well, and samples were enclosed with nylon membrane coverlids. The samples were incubated 1 h at 37°C free CO_2_. XF24 sensor cartridges were hydrated using XF24 calibrant overnight at 37°C free CO_2_. FCCP concentration was optimized by using different concentrations in the ports [1 µM (stock 10 µM) and 10 µM (stock 100 µM)]. For the ATP rate assay, Port A had 25 µM oligomycin (stock, 250 µM; Sigma-Aldrich), Port B had 10 µM FCCP (stock, 100 µM; Sigma-Aldrich), Port C had 10 µM FCCP (stock, 100 µM; Sigma-Aldrich), and Port D had 27 µM rotenone (stock, 270 µM; Sigma-Aldrich) plus 45 µM antimycin A (stock, 450 µM; Sigma-Aldrich). These compounds modulate the activity in the mitochondrial respiration ETS and Complex V or ATP synthase. The system recorded oxygen flux values indicate only ETS oxygen consumption. Rotenone and antimycin A inhibited Complex I and Complex III, respectively, stopping the mitochondrial respiration, FCCP was an ionophore showing maximal capacity of the ETS, and oligomycin generates information of oxygen consumption devoted to ATP production via blocking the *F*_o_ subunit of the ATP synthase. The program was run at 37°C. Data were analyzed using the WAVE software (Agilent Technologies) and normalized based on the routine respiration values (Ortiz-Rodriguez et al., 2024).

#### ABSs

The adult brain cortex was sliced using a microtome (Compresstome VF-300, Precisionary Instruments; Weidinger et al., 2013), following the protocol previously described to a thickness of 200 µm. Slices were cultured floating in 50% MEM + HEPES no glutamine (Thermo Fisher Scientific) supplemented with 25% horse serum heat inactivated (Thermo Fisher Scientific), 25% Hank's solution (Thermo Fisher Scientific), 6.5 mg/ml of glucose (Fluka BioChemika), and penicillin/streptomycin mixture (Thermo Fisher Scientific), pH 7.2, medium for 1 h, at 37°C and 5% CO_2_. Afterward, the inhibitors and glutamate were prepared in a 1 mM CaCl_2_ (Sigma-Aldrich) and 0.22 mM l-cysteine hydrochloride (Sigma-Aldrich) 1× PBS buffer (Sigma-Aldrich). Samples were exposed to the pretreatments 10 µM ferrostatin-1 [Fer-1, Eurobio Scientific (Li et al., 2017)], 200 µM deferoxamine [DFx; Sigma-Aldrich (Donneys et al., 2013)], and 10 µM rosiglitazone [(Rosi, Cayman Chemical (Wong et al., 2015)] for 30 min, and 0.9 mM of glutamate [Sigma-Aldrich (Mark et al., 2001)] was added for 20 h. Samples were incubated at 37°C in a humidified atmosphere. At least three slices were included per experiment. Each slice, regardless of which rat it was obtained from, was treated as an independent sample.

#### Lactate dehydrogenase release

Lactate dehydrogenase (LDH) release from supernatant and brain slice homogenate was measured separately following the protocol published in Vaglio-Garro et al. (2024a).

#### Oxoglutarate dehydrogenase activity

OGDHC activity in the postnatal brain slice homogenate was measured following the protocol described in Weidinger et al. (2023).

#### Nitric oxide quantification

Nitric oxide (NO) was measured with Nitric Oxide Analyzer (NOA 280i; General Electric, Boulder, CO, USA) by reduction of the sample and measurement of the potential difference (millivolts) caused by the reduction from nitrite to nitic oxide by sodium iodide and acetic acid. The measurement chamber was cleaned and filled with 8 ml of sodium iodide reduction solution [1% sodium iodide + 3 ml H_2_O_dd_ + 30 ml acetic acid (99, 7%)]. After calibration with nitrogen solution, detection of NO was done in 100 µl of the postnatal brain homogenate. The obtained peaks correlating for the potential difference were then integrated, and the obtained areas of the different treatments were compared with determine changes in NO levels caused by the treatments.

#### Histological staining

Postnatal organotypic brain slices were immersed in 4% formaldehyde (VWR Chemicals BDH) for 24 h on agitation. Samples were rinsed, dehydrated in increasing series of ethanol concentration, and embedded in paraffin with the Tissue processor HP3000 (Dakewe Medical); 4 μm sections were cut with a microtome (Microm HM 355 S, Thermo Fisher Scientific), placed on glass slides and dried overnight at 37°C. Prior to all staining, sections were deparaffinized and rehydrated. They were stained with hematoxylin and eosin (HE) following standard procedures. Imaging was performed on a Nikon microscope with a DS-FI3 camera (Nikon Instruments) with a 10× objective magnification. The images were acquired in the NIS-Elements software (version 5.21.00, Nikon Instruments).

#### LPO measurements

LPO was measured in all the samples after adding all the different treatments in a live staining with BODIPY 581/591 C11 (Invitrogen) adapting the protocol described in Smirnova et al. (2025). Oxidized BODIPY in living cells, shifts the emission peak from ∼590 to ∼510 nm. The samples were stained with 5 µM of BODIPY for 45 min at 37°C following with a washed of PBS 1×. Imaging was performed by a Zeiss LSM510 Meta laser scanning confocal microscope (Zeiss), with ex/em of 581/591 nm to detect nonoxidized state and ex/em of 488/510 nm for oxidized state. Pictures were processed with ImageJ/Fiji (version 2.16.0/1.54p, National Institutes of Health). The ratio nonoxidized/(nonoxidized + oxidized) was calculated to determine LPO.

#### Statistical analysis

Brain cortex samples were obtained by sectioning the cortex into multiple slices. Because each slice originated from a different cortical region, the slices were not considered technical replicates. Instead, they were treated as repeated measurements, and all measurements were included in the statistical analysis, regardless of the animal from which they were collected. Five total animals were used for this experiment.

The exception was the experiments with ABS, in which we aimed to determine LPO and LDH release from the same well. In these experiments, we averaged the fluorescence measurements from two slices within each well and determined LDH release from the same two slices.

Regarding outlier analysis, we first applied the ROUT Q test. If this test identified no outliers or a single outlier per group, we subsequently applied the Grubbs test and excluded the respective outlier(s). The results of the ROUT Q test were used only for one graph, namely, Figure 2*E*.

Samples analyzed on the same plate were considered matched samples, and the appropriate statistical analysis—repeated-measures ANOVA (RM-ANOVA) or a mixed-effects model (REML)—was selected by the software.

In contrast, measurements performed on the same brain homogenate, which represents a uniform mixture with a constant composition of brain regions, were considered technical replicates. For statistical analysis, the average value of these technical replicates was used.

All data are presented as mean ± SEM. The method used for statistical evaluation is indicated in figure legends. The evaluation was performed using GraphPad Prism 10.1.2. Outliers were identified and removed with ROUT *Q* = 1%. A part of the data was normalized against the control values and converted to percentages.

## Results

First, we determined the enzymatic activities of malate dehydrogenase (MDH), PDHC, GDH, and OGDHC (also known as ketoglutarate dehydrogenase complex, KGDHC) enzymes which participate in the mitochondrial oxidative metabolism of pyruvate and glutamate, respectively (Fig. 1*A*–*C*). Under nonstarving physiological conditions, the major acetyl-CoA source for the TCA cycle is pyruvate (Fig. 1*A*), while glutamate predominantly circulates in glutamate–glutamine cycle (McKenna et al., 2016; Sonnewald and Schousboe, 2016). However, if the electron supply from pyruvate is not sufficient or the concentration of cytoplasmic pool of glutamate is elevated, glutamate can also be used for energy purposes (Fig. 1*B*). Feeding the TCA cycle, both pyruvate and glutamate, delivers NADH to the mitochondrial ETS (Fig. 1*C*). In contrast to PDHC and OGDHC, MDH is remarkably expressed in both cell types. Using this advantage, we quantified the contributions of the pyruvate and glutamate metabolic pathways in isolated astrocytes and brain homogenates, using MDH as a reference enzyme. Otherwise, it is difficult to make this comparison directly, because it is not possible to isolate all astrocytes and all mitochondria from a single brain tissue. These data (Fig. 1*D*) show that the contribution of OGDHC/PDHC located in astrocytes is marginal (<1%) compared with total enzymatic activity of these enzymes in the entire brain mitochondria. This suggests that the glutamate or pyruvate-induced mitochondrial respiration (as well as ATP synthesis) originates almost exclusively from neurons. Based on this fact, we performed the following experiments assuming that the respiration observed in the brain tissue primarily originates from neuronal mitochondria.

**Figure 1.**
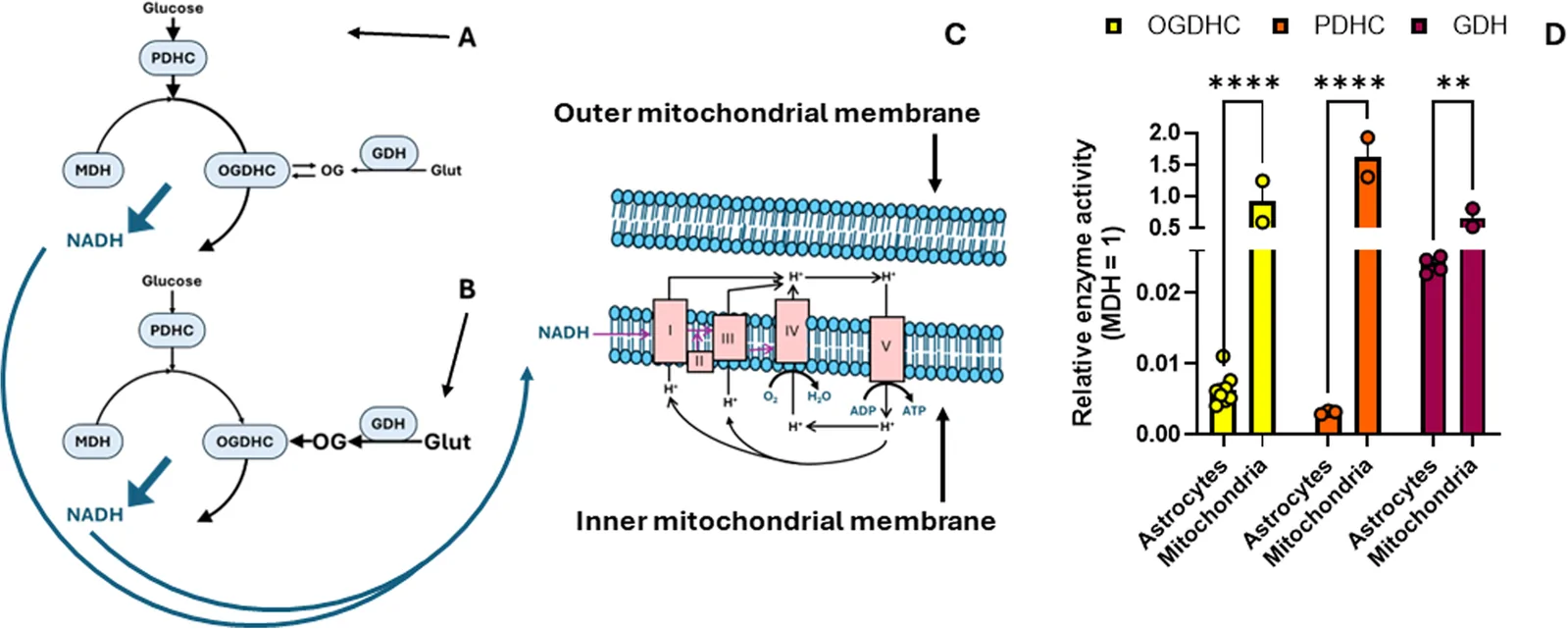
***A***, Mitochondrial respiration with pyruvate (synthetized from glucose by PDHC). ***B***, Mitochondrial respiration with glutamate. ***C***, Transport electron system of mitochondria. ***D***, The activities of OGDHC, PDHC, and GDH in primary astrocytes and in isolated brain mitochondria. The activities were normalized to MDH. The results show that in astrocytes, pyruvate and glutamate pathways are of marginal importance (0.006 and 0.004, respectively), compared with brain mitochondria (0.9 and 1.8, respectively). This is in line with the knowledge that these energy conservation pathways are predominantly located in neurons and <1% in astrocytes. The experimental timeline is shown in Extended Data Figure 1-1. Statistics: Data are displayed as mean ± SEM and evaluated by ANOVA/Sidak's multiple-comparisons test. Significance **p < 0.001; ****p < 0.00001. Abbreviations: PDHC, pyruvate dehydrogenase complex; MDH, malate dehydrogenase; OGDHC, oxoglutarate dehydrogenase; OG, oxoglutarate; Glut, glutamate; GDH, glutamate dehydrogenase.

10.1523/ENEURO.0439-25.2026.f1-1 Figure 1-1 **Timeline of postnatal brain slice experiment.** Organotypic brain slices, obtained from sagittal slicing from Sprague Dawley pups’ brains, samples were cultured for 14 days. Samples were pre-treated with inhibitors for 30 min, later samples were treated only with glutamate for 2 h. Fresh medium was added to the samples, and they were incubated on it for 24 h. Later, supernatant was collected and cooled down in liquid nitrogen to measure lactate dehydrogenase (LDH) and nitric oxide (NO). Slices were either punched to measure oxygen consumption or homogenized to measure oxygen consumption in Oroboros O2k or oxoglutarate dehydrogenase complex (OGDHC) activity. Download Figure 1-1, TIF file.

Our next aim was to investigate the effect of glutamate toxicity on mitochondrial respiration on brain slices along with effects of the inhibitors of glutamate receptors and ferroptosis, which are supposed to modulate cell death pathways. Mitochondrial respiration in nonhomogenized brain slices was assessed using the Seahorse Metabolic Analyzer, with one slice punched placed per each well. We measured the routine respiration [RR; also called basal respiration (BR)], ATP-linked respiration (ATP-R), and uncoupled respiration (UC-R; Fig. 2*A*). Effect of glutamate, memantine, and ferrostatin-1 on the ratio of oxygen flux of ATP synthesis-linked respiration to extracellular acidification rate (ECAR) is displayed in Figure 2*B*. The data show that oxygen flux of ATP synthesis-linked respiration to ECAR is significantly increased in all glutamate groups.

**Figure 2.**
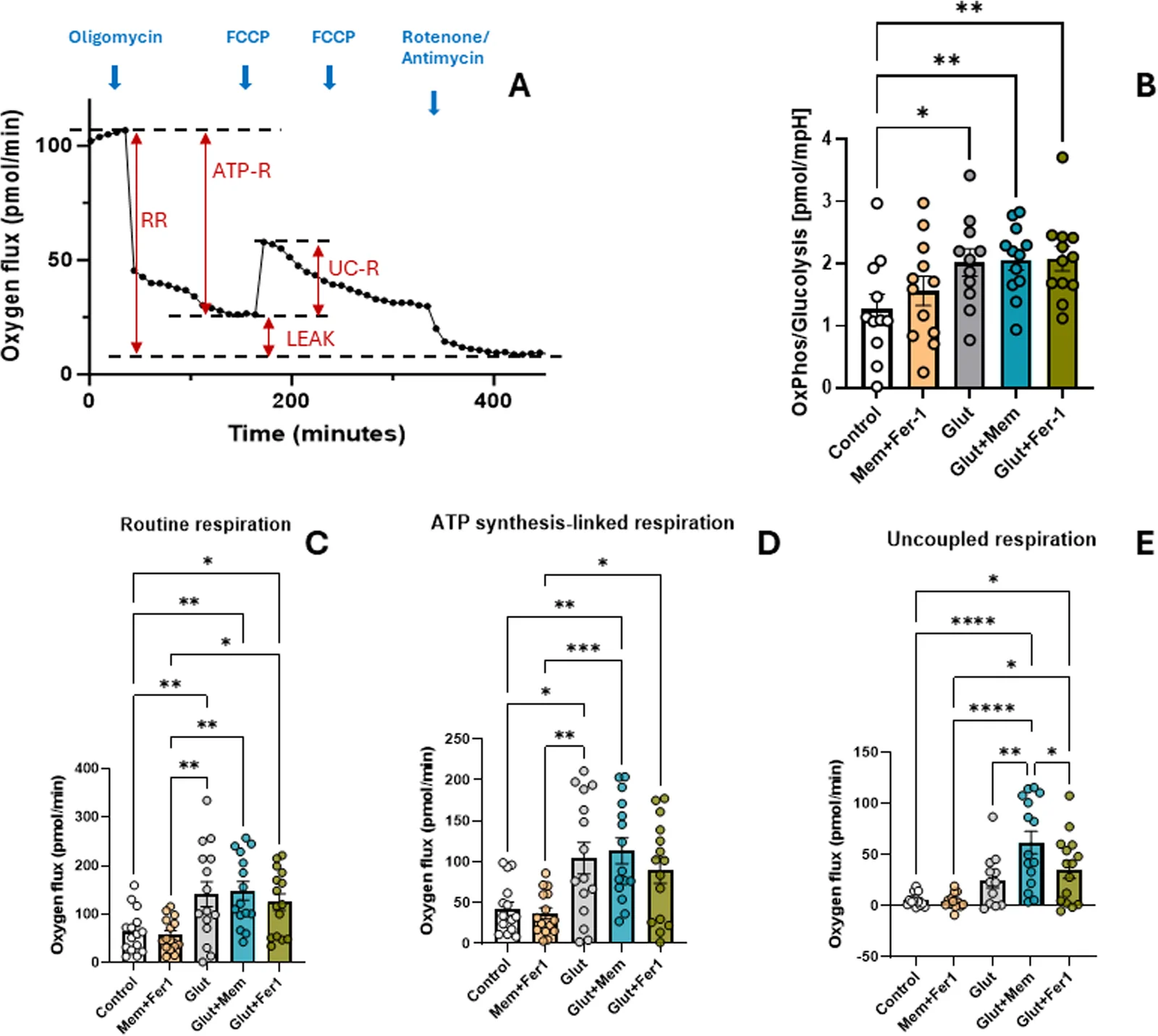
Effect of glutamate, memantine, and ferrostatin-1 on the mitochondrial respiration in nonhomogenized BSC after 24 h. Samples were pretreated with memantine or ferrostatin-1 for 30 min and treated with glutamate for 2 h. ***A***, Trace of oxygen flux and its analysis. ***B***, Effect of glutamate, memantine, and ferrostatin-1 on the ratio of oxygen flux of ATP synthesis-linked respiration to ECAR. An increase in this ratio shows that metabolic preference in groups treated with glutamate is shifted in favor of aerobic respiration, all groups with glutamate (Glut and Glut + Mem, Glut + Fer-1) present an increased ratio. ***C***, Quantification of routine respiration. ***D***, Quantification of ATP synthesis-linked respiration. ***E***, Quantification of uncoupled respiration. Oxygen flux was determined in picomole of oxygen consumed per minute by one BSC. Independent experimental traces are shown in Extended Data Figure 2-1. Statistics: Data are displayed as mean ± SEM and evaluated by ANOVA/Sidak’s multiple-comparisons test. Significance: * *p* < 0.05, ** *p* < 0.01, *** *p* < 0.001, **** *p* < 0.0001. Abbreviations: Glut, glutamate; RR, routine respiration; ATP-R, ATP synthesis-linked respiration; UC-R, uncoupled respiration; FCCP, carbonylcyanid-*p*-trifluormethoxyphenylhydrazone; Mem, memantine; Fer-1, ferrostatin-1; BSC, brain slice cultures.

10.1523/ENEURO.0439-25.2026.f2-1 Figure 2-1 **Traces of oxygen flux in four independent experiments with organotypic brain slices.** The traces were recorded with Seahorse as described in the Methods section. **Abbreviations:** Glut: glutamate, Fer-1: ferrostatin-1, Mem: memantine. Download Figure 2-1, TIF file.

Metabolic analysis of the samples using glutamate exhibited a substantially elevated respiration rate. This increase was observed in routine (Fig. 2*C*), ATP synthesis-linked respiration (Fig. 2*D*), and uncoupled respiration (Fig. 2*E*), except for uncoupled respiration in the Glut group, which did not differ from the control (Fig. 2*E*).

To further characterize the metabolic status of the cells before and after glutamate treatment, we calculated the ratio of oxygen flux of ATP synthesis-linked respiration to ECAR (Extended Data Fig. 2-1). ECAR characterizes the rate of glycolysis. The ratio was substantially increased in all groups containing glutamate (Glut and Glut + Mem, Glut + Fer-1) suggesting that the presence of glutamate shifts the metabolic preference of cells treated with glutamate in favor of aerobic respiration.

Previous studies have shown that elevated levels of glutamate may upregulate the activity of OGDHC and that endogenous NO serves as a posttranslational regulator of OGDHC and may affect toxic effects of glutamate (Weidinger et al., 2023). To investigate whether this mechanism contributes to the acceleration of aerobic metabolism in our model, we assessed both OGDHC activity and NO levels. Figure 3 shows that OGDHC activity (Fig. 3*A*,*B*) and NO levels (Fig. 3*C*,*D*), in brain homogenate of BSC, did not change significantly after memantine pretreatment in control, Mem and Glut + Mem groups; OGDHC activity tended to be higher in the Glut group compared with the Mem group. These results suggest that either the rate of the TCA cycle is elevated in the presence of glutamate or that the respiration is limited by endogenous concentrations TCA cycle substrates, which is compensated by increased transport of glutamate into the cells in the Glut groups. To test this hypothesis, we homogenized brain slices before measuring mitochondrial respiration in the presence of high glutamate concentrations.

**Figure 3.**
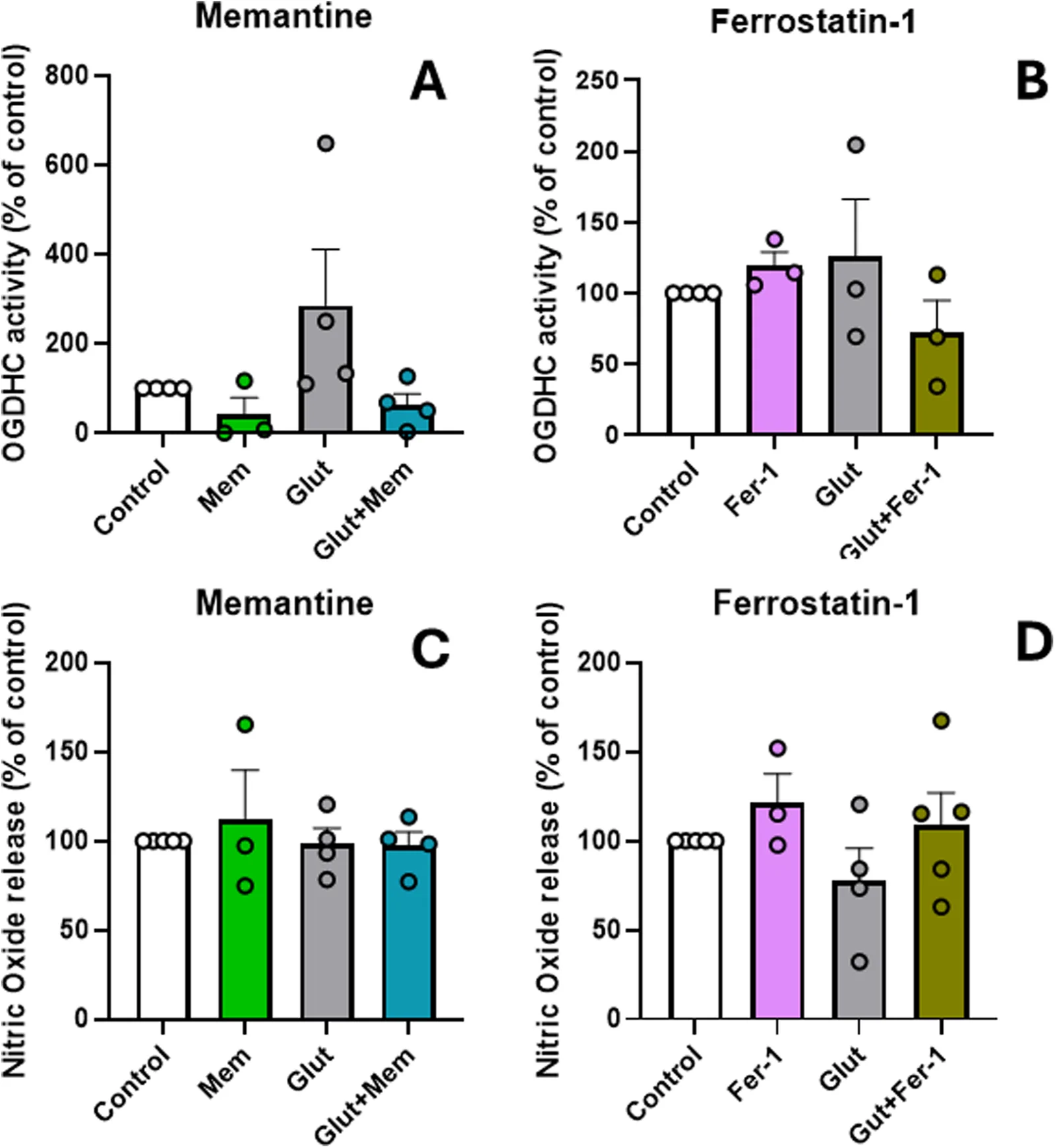
OGDHC activity (***A, B***) and NO released (***C, D***) of BSC after 24 h incubation. Samples were pretreated with inhibitors for 30 min and treated with glutamate for 2 h. ***A***, Enzymatic activity after inhibiting glutamate excitotoxicity with memantine. ***B***, Enzymatic activity after inhibiting ferroptosis with ferrostatin-1. ***C***, NO release after inhibiting glutamate excitotoxicity with memantine. ***D***, NO release after inhibiting ferroptosis with ferrostatin-1. Values were normalized with control only for OGDHC experiments and expressed in percentage. Details are provided in Materials and Methods. Statistics: Data are displayed as mean ± SEM and evaluated by ANOVA/Sidak’s multiple-comparisons test. Significance *p < 0.05. Abbreviations: Glut, glutamate; Fer-1, ferrostatin-1; Mem, memantine; OGDHC, oxoglutarate dehydrogenase complex; NO, nitric oxide; BSC, brain slice cultures.

Figure 4*A* shows a typical oxygen uptake trace in the chamber containing homogenate of three slices. After each titration, the linear part of the oxygen consumption trace was selected, and the slope was determined, as shown below the corresponding portion of the trace.

**Figure 4.**
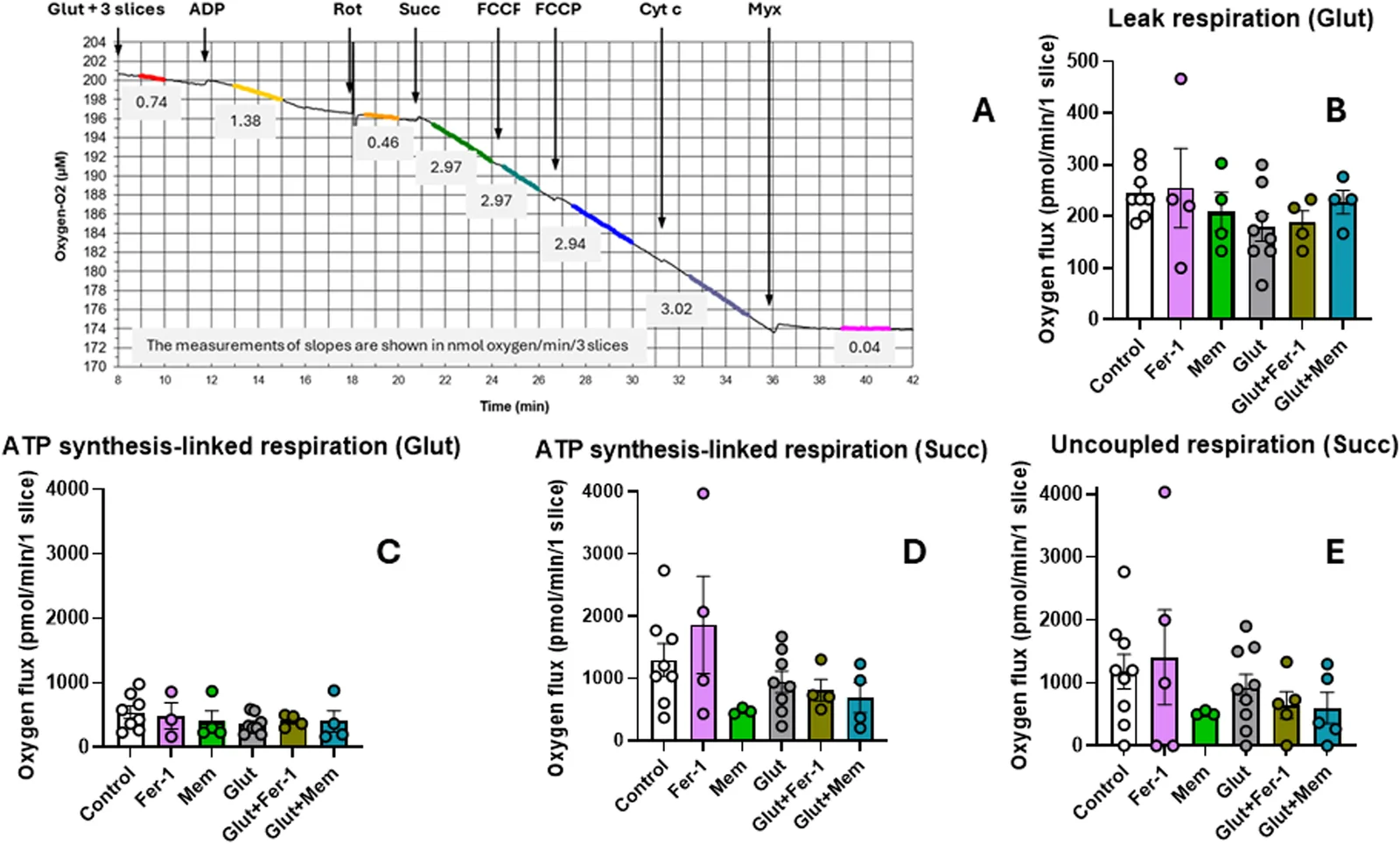
Effect of glutamate, memantine, and ferrostatin-1 on the mitochondrial respiration of brain homogenate from three BSC after 24 h incubation. Samples were pretreatment with memantine or ferrostatin-1 for 30 min and treated with glutamate for 2 h. ***A***, Analysis of the trace of oxygen flux. ***B***, State 2 (leak) respiration with glutamate. ***C***, State 3 (ATP synthesis- linked) respiration with glutamate. ***D***, State 3 (ATP synthesis-linked) respiration with succinate. ***E***, Uncoupled respiration. Injection of cyt c did not show significant chances in the respiration rate (data not shown). Oxygen flux shows the consumption of three brain slices in nanomoles of oxygen per minute; data were recalculated in picomoles per minute per slice. Details are provided in Materials and Methods. Statistics: Data are displayed as mean ± SEM and evaluated by ANOVA/Sidak’s multiple-comparisons test. No significant changes were determined. Abbreviations: Glut, glutamate; Fer-1, ferrostatin-1; Mem, memantine; FCCP, carbonylcyanid-*p*-trifluormethoxyphenylhydrazone; Rot, rotenone; Succ, succinate; BSC, brain slice cultures.

In contrast to the analysis of nonhomogenized slices, we did not observe any increase in the mitochondrial respiration rate in homogenized slices (Fig. 4*B*–*E*). Interestingly, the respiration in the glutamate-treated groups showed a nonsignificant trend toward a decrease. This trend was evident in ATP-linked respiration with glutamate (Fig. 4*C*) and succinate (Fig. 4*D*), as well as in uncoupled respiration (Fig. 4*E*), but not in routine respiration (Fig. 4*B*). The Seahorse Metabolic Analyzer's signal depends on the slice position and thickness, whereas in the Oroboros O2k, tissue homogenates are evenly distributed in the chamber due to continuous stirring and can be normalized not only to the number of slices but also to the weight of homogenized slices.

This normalization did not substantially alter the results (Fig. 5*A*–*D*). Of note, the lowest levels of leak and ATP-linked respiration were observed in the glutamate and glutamate + memantine groups, whereas, in the presence of ferrostatin-1, mean oxygen flux values reached levels above those of the control group for both leak and ATP synthesis-linked respirations. Ferrostatin-1 significantly increased leak respiration (Fig. 5*A*) and tended to increase ATP-linked respiration compared with the Glut group (Fig. 5*B*). These data suggest a slight impairment of mitochondrial function following glutamate exposure, which was partially restored by ferrostatin-1. We hypothesize that this effect may reflect a beneficial action of ferrostatin-1 in our model.

**Figure 5.**
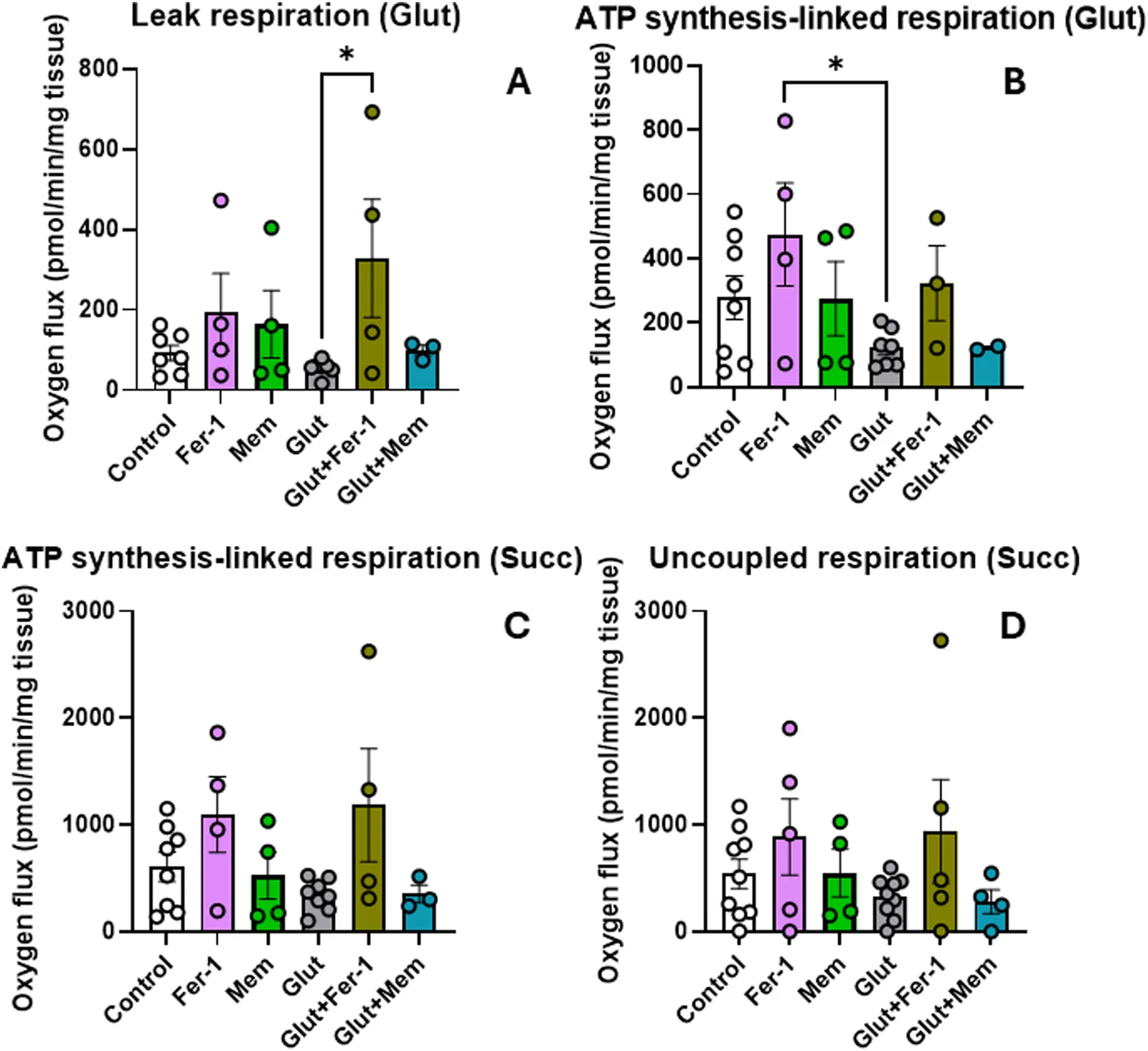
Effect of glutamate, memantine, and ferrostatin-1 on the mitochondrial respiration of homogenized of three BSC after 24 h incubation. Samples were pretreatment with memantine or ferrostatin-1 for 30 min and treatment with glutamate for 2 h. ***A***, State 2 (leak) respiration with glutamate. ***B***, State 3 (ATP synthesis) respiration with glutamate. ***C***, State 3 (ATP synthesis) respiration with succinate. ***D***, Uncoupled respiration. Oxygen flux was determined in nanomoles of oxygen per minute; data were recalculated into picomoles per minute per milligram of tissue. Details are provided in Materials and Methods. The effect of AP5 and CNQX is shown in Extended Data Figure 5-1. Statistics: Data are displayed as mean ± SEM and evaluated by ANOVA/Sidak’s multiple-comparisons test. No significant changes were determined. Abbreviations: Glut, glutamate; Fer-1, ferrostatin-1; Mem, memantine; FCCP, carbonylcyanid-*p*-trifluormethoxyphenylhydrazone; Rot, rotenone; Succ, succinate; BSC, brain slice cultures.

10.1523/ENEURO.0439-25.2026.f5-1 Figure 5-1 **Effect of AP-5** **+** **CNQX on the oxygen flux in the presence of glutamate in non-homogenized BSC** (**A, B**) **and in homogenized BSC** (**C, D**). **A**. Routine respiration **B.** ATP synthesis-linked respiration. **C**. Leak respiration. **D**. ATP synthesis-linked respiration. **Statistics:** Data are displayed as mean ± SEM and evaluated by Kruskal Wallis Uncorrected Dunn test. No significant differences were determined. **Abbreviations:** Glut: glutamate, AP-5: (2R)-amino-5-phosphonopentanoate, CNQX: cyanquixaline, ATP: adenosine triphosphate, BSC: brain slice cultures. Download Figure 5-1, TIF file.

To test this hypothesis further, we assessed cell death in the experimental groups by measuring LDH release. The release of LDH was significantly higher following glutamate treatment (Fig. 6*A*,*B*). Memantine had no significant effect on glutamate-induced cell death (Fig. 6*A*), whereas ferrostatin-1 substantially decreased LDH release (Fig. 6*B*). These results support our hypothesis that inhibition of ferroptosis by ferrostatin-1 may have a protective effect. Neither memantine nor ferrostatin-1 affected LDH release in control samples. The lack of a beneficial effect of memantine was surprising, as we previously observed its protective effect in neuronal cultures.

**Figure 6.**
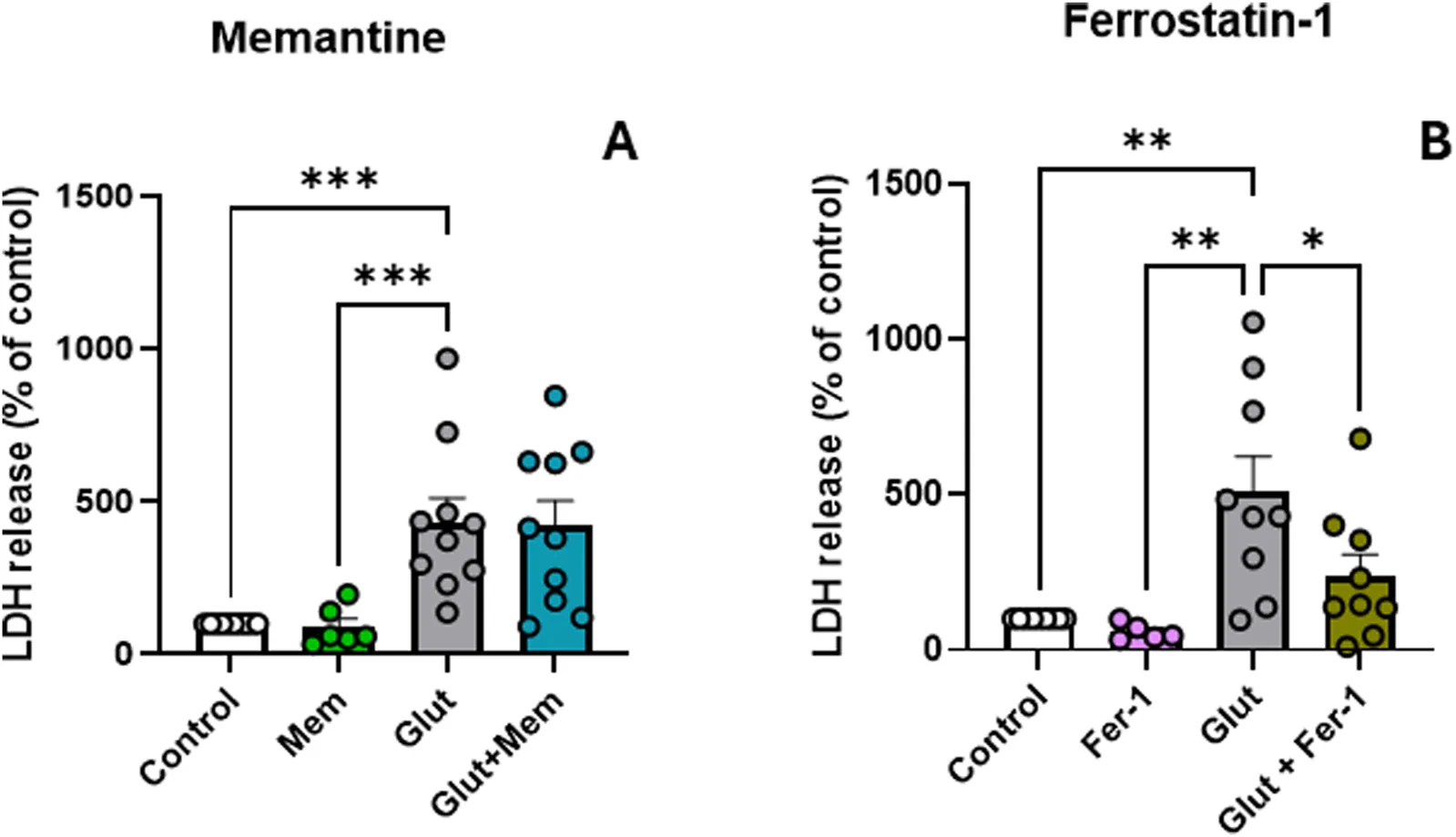
LDH released of BSC after 24 h incubation. Samples were pretreatment with memantine or ferrostatin-1 for 30 min and treated with Glut for 2 h. ***A***, Inhibition of glutamate excitotoxicity cell death pathway with memantine. ***B***, Inhibition of ferroptosis cell death pathway with ferrostatin-1. Values were normalized with control and expressed in percentage. The effect of AP5 and CNQX are shown in Extended Data Figure 6-1. Statistics: Data are displayed as mean ± SEM and evaluated by ANOVA/Sidak’s multiple-comparisons test. Significance **p < 0.01; ***p < 0.001; ns, not significant. Abbreviations: Glut, glutamate; Fer-1, ferrostatin-1; Mem, memantine; BSC, brain slice cultures; LDH, lactate dehydrogenase.

10.1523/ENEURO.0439-25.2026.f6-1 Figure 6-1 **LDH released by BSC after 24 h incubation.** Samples were pre-treated with AP5 + CNQX for 30 min and treated with glutamate for 2 h. **Statistics:** Data are displayed as mean ± SEM and evaluated by Kruskal Wallis Uncorrected Dunn test. No significant differences were observed. **Abbreviations:** LDH: Lactate dehydrogenase, Glut: glutamate, AP-5: (2R)-amino-5-phosphonopentanoate, CNQX: cyanquixaline, ATP: adenosine triphosphate, BSC: brain slice cultures. Download Figure 6-1, TIF file.

To further investigate the impact of glutamate-induced excitotoxicity, we tested two other glutamate NMDA receptor antagonists, (2R)-amino-5-phosphonopentanoate (AP-5) and cyanquixaline (CNQX). The effects of mitochondrial respiration between the AP-5 + CNQX and Glut groups were not significant (Extended Data Fig. 5-1*A*,*B*). We observed in the BSC homogenates, for leak respiration and ATP synthesis-linked oxygen flux, higher mean values for AP-5 + CNQX compared with the Glut group, but the differences were not significant (Extended Data Fig. 5-1*C*,*D*). Finally, we assessed the effects of AP-5 + CNQX on glutamate toxicity by measuring LDH release (Extended Data Fig. 6-1). Summarizing, no significant differences were observed between the inhibitors (AP5 + CNQXa) and the Glut groups. These results suggest that ferroptosis is a cell death mechanism occurring in our model.

To further support the activation of ferroptosis and the potential protective effect of ferrostatin-1, we repeated the experiments described above using brain slices that were 100 µm thicker, allowing for histological examination. Due to technical limitations, we were unable to obtain thinner brain slices. The histological slices were stained with hematoxylin and eosin.

Histological analysis revealed that control samples (Fig. 7*A*,*D*) contained uniform neurons with pale cytoplasm and dark nuclei, indicative of healthy, intact neurons. Upon glutamate treatment (Fig. 7*B*,*E*), we observed cells with eosinophilic cytoplasm, shrunken, hyperchromatic (pyknotic) nuclei, or cells without nuclei; quantification can be seen in Table 1. Treatment with ferrostatin-1 ameliorated some of the changes induced by glutamate (Fig. 7*C*,*F*), with more neurons exhibiting pale cytoplasm and dark nuclei.

**Figure 7.**
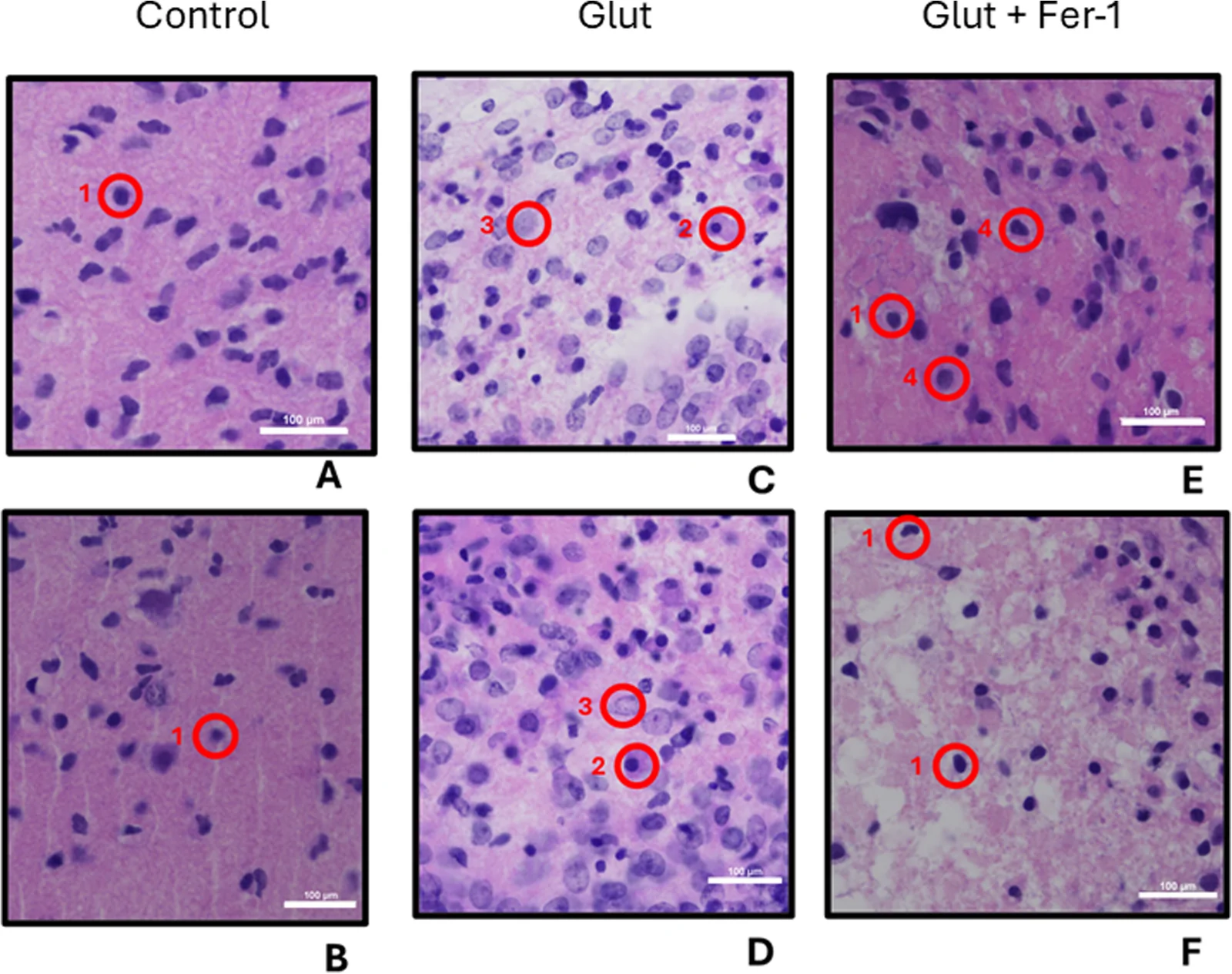
Effect of glutamate and ferrostatin-1 on the morphology of the cells of BSC stained with hematoxylin eosin. ***A***, ***B***, Control. ***C***, ***D***, Glut. ***E***, ***F***, Glut + Fer-1. The red circles are surrounding some examples. 1, Uniform neurons with pale cytoplasm and dark nuclei (in control). 2, Deeply pink (eosinophilic) cells with shrunken, hyperchromatic (pyknotic) nuclei. 3, Cells without nuclei. 4, Less advanced eosinophilic cells. Scale bar, 100 µm. Abbreviations: Glut, glutamate; Fer-1, ferrostatin-1; BSC, brain slice cultures.

**Table 1.** Quantification of histological images shown in Figure 7

	Treatment		
	Control	Glutamate	Glutamate + ferrostatin-1
Uniform cells	58/58 (A) + 50/50 (B) Average 54/54	23/84 C 18/92 (D) Average 20.5/88	52/66 (E) 15/39 (F) Average 33.5/52
Shrunken cells	0/58 (A) 0/50 (B) Average 0/54	21/84 (C) 50/92 (D) = Average 35.5/88	0/66 (E) 12/39 (F) Average 6/52
Cells without nuclei	0/58 (A) 0/50 (B) Average 0/54	40/84 (C) 24/92 (D) Average 32/88	0/66 (E) 0/39 (F) Average 0/52
Less advanced eosinophilic cells	0/58 (A) 0/50 (B) Average 0/54	0/84 (C) 0/92 (D) Average 0/88	14/66 (E) 12/39 (F) Average 13/52

The data are presented as [specific cell number]/[total cell number] (image number) and average number of specific cells.

However, we also observed eosinophilic cells, which were less advanced than those in the glutamate-only group. In these cells, only a portion of the cell body was eosinophilic, suggesting that ferrostatin-1 partially protected against glutamate-induced toxicity. The quantification of histological images is shown in Table 1.

To further support our conclusion that ferroptosis is one of the major cell death pathways activated by glutamate toxicity in organotypic brain cultures (Fig. 6), we performed additional experiments using ABSs and tested several compounds targeting key components of the ferroptosis pathway.

As in the organotypic brain cultures, we evaluated the effects of ferrostatin-1. In addition, we tested rosiglitazone, an inhibitor of acyl-CoA synthetase long-chain family member 4 (ACSL4), an enzyme that catalyzes the esterification of arachidonic and adrenic acids preparing them for synthesis of phospholipids, enriching membranes with oxidizable polyunsaturated fatty acids and thereby promoting the LPO that drives ferroptosis (Ding et al., 2023). We also investigated effect of deferoxamine, a well-established iron chelator, which was shown to inhibit LPO and ferroptosis in several experimental models of brain diseases (Costa et al., 2023).

In ABSs, we also quantified LPO, a hallmark and essential feature of ferroptosis. The typical images obtained in this study are shown in the Extended Data Figure 8-1. These experiments yielded clear results (Fig. 8). After 20 h of glutamate exposure, ABSs exhibited a marked increase in LPO. Glutamate-induced LPO was completely suppressed by ferrostatin-1, rosiglitazone, and deferoxamine. This indicates that the ferroptotic pathway was activated also in ABSs.

**Figure 8.**
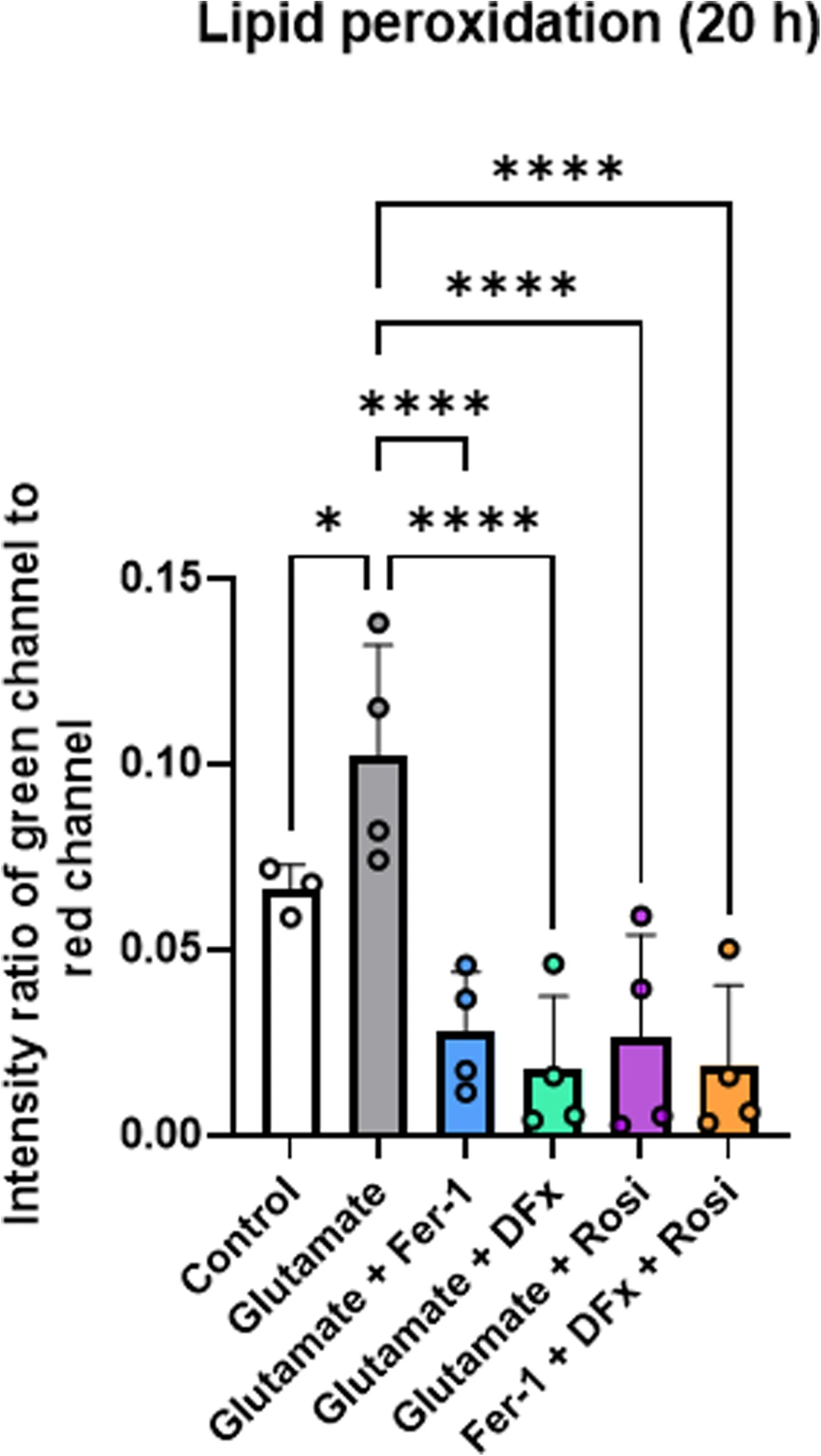
Quantification of LPO. Green/red fluorescence ratio of C11-BODIPY 581/591. Treatment groups: Control, 0.9 mM glutamate, 0.9 mM glutamate + 10 µM Fer-1, 0.9 mM glutamate + 200 µM DFx, 0.9 mM glutamate + 10 µM Rosi, and 10 µM Fer-1 + 200 µM DFx + 10 µM Rosi. Each point represents a value obtained from two slices incubated in one well, average of 6–8 images. The confocal images are shown in Extended Data Figure 8-1. Statistics: Data are displayed as mean ± SEM and evaluated by RM-ANOVA/Holm–Šidák test. *p < 0.05, ****p < 0.0001. Abbreviations: DFx, deferoxamine mesylate; Fer-1, ferrostatin-1; Glut, glutamate; Rosi, rosiglitazone.

10.1523/ENEURO.0439-25.2026.f8-1 Figure 8-1 **A.** Representative confocal fluorescence images of the reduced C11-BODIPY 581/591 (red channel) stained adult rat brain slices following the indicated treatments. **B.** Representative confocal fluorescence images of the oxidized C11-BODIPY 581/591 (green channel) in the corresponding brain slices. Scale bar = 200 µm. The green channel brightness was uniformly adjusted across all images using ImageJ/Figi by setting the minimum display intensity value to 16 to improve visualization. The red channel was not adjusted. Brightness and contrast adjustments were applied solely for visualization purposes and do not reflect changes made during quantitative fluorescence analysis. Treatment groups: Control, 0.9 mM glutamate, 0.9 mM glutamate + 10 µM Fer-1, 0.9 mM glutamate + 200 µM DFx, 0.9 mM glutamate + 10 µM Rosi, and 10 µM Fer-1 + 200 µM DFx + 10 µM Rosi. **Abbreviations:** DFx: deferoxamine mesylate, Fer-1: ferrostatin-1, Rosi: rosiglitazone. Download Figure 8-1, TIF file.

Next, we evaluated the effects of these compounds on glutamate-induced cell death by measuring LDH release. Consistent with the LPO data, the treatments with ferrostatin-1 or with rosiglitazone significantly reduced glutamate-induced cell death (Fig. 9). In contrast, despite its pronounced inhibitory effect on LPO, deferoxamine did not provide significant protection against glutamate-induced cell death (Fig. 9).

**Figure 9.**
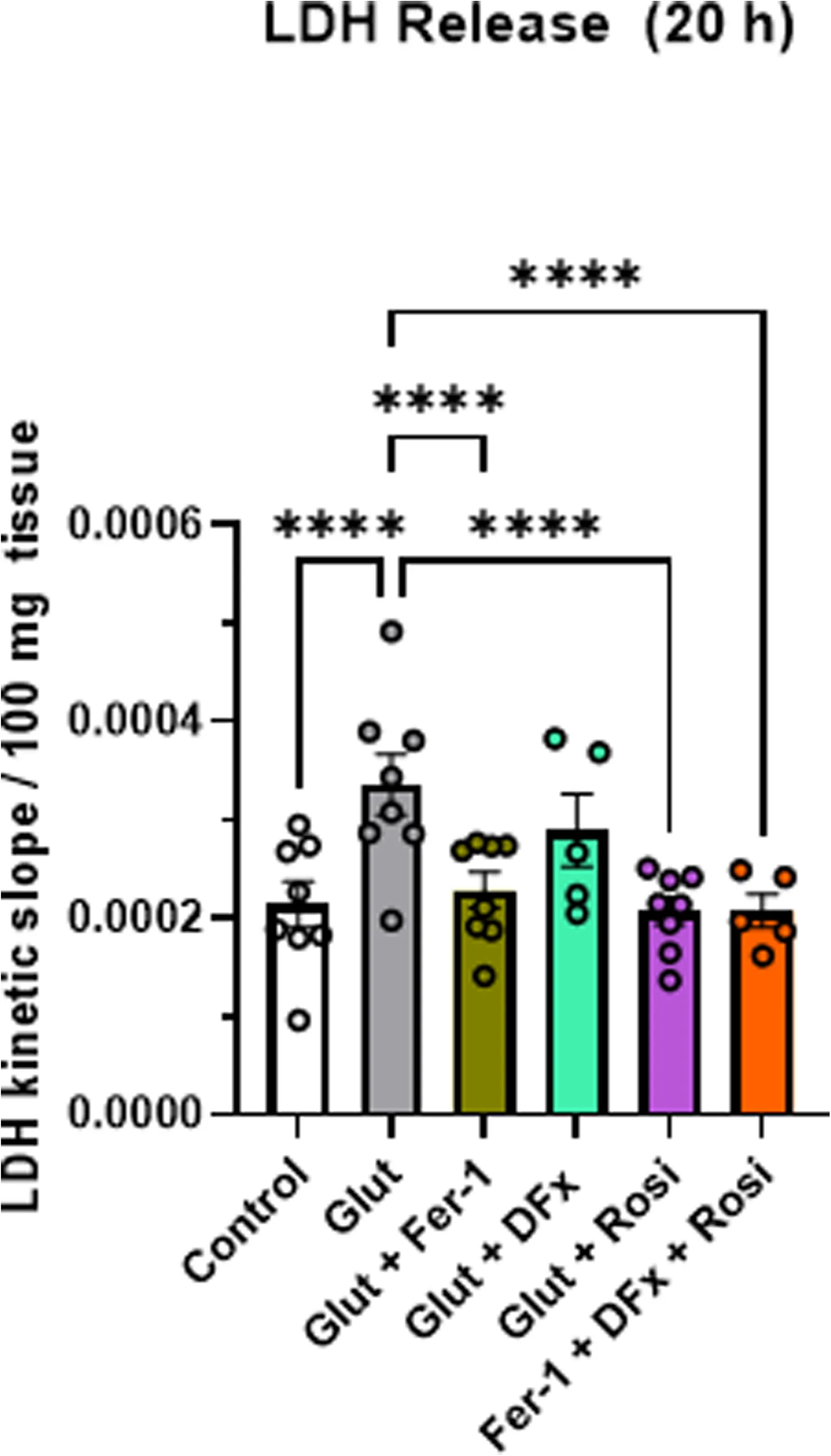
Effect of glutamate, ferrostatin-1, deferoxamine, and rosiglitazone on LDH release of BSC after 20 h incubation. Treatment groups: Control, 0.9 mM glutamate, 0.9 mM glutamate + 10 µM Fer-1, 0.9 mM glutamate + 200 µM DFx, 0.9 mM glutamate + 10 µM Rosi, and 10 µM Fer-1 + 200 µM DFx + 10 µM Rosi. Each point represents LDH levels obtained from one well containing three slices. Statistics: Data are displayed as mean ± SEM and evaluated by RM-ANOVA/Holm–Šidák multiple-comparisons test. **p < 0.01. Abbreviations: Glut, glutamate; DFx, deferoxamine mesylate; Fer-1, ferrostatin-1; Rosi, rosiglitazone; LDH, lactate dehydrogenase.

## Discussion

The aim of this work was to find a link between mitochondrial function/dysfunction and cell death in organotypic brain slices culture exposed to toxic concentration of glutamate. First, we estimated the contribution of different brain cell types to the respiration determined in the brain tissue with emphasis on glutamate as a mitochondrial respiration substrate. Among all types of brain cells, only neurons and astrocytes are predominantly involved in glutamate metabolism. In this study, we estimated the relative contribution of astrocytes in the oxidative metabolism of the brain tissue, and our results indicate it is <1%. We concluded that ∼99% of glutamate is converted to ATP in neurons (Fig. 1). Given that ATP synthesis is linked to oxygen consumption, we measured the cellular respiration of brain slices as a method to characterize mitochondrial function in neurons.

Next, we tested the nonhomogenized BSC in order to examine how they respond to glutamate. In accordance with previous experience (Weidinger et al., 2023), we expected to observe a drop in mitochondrial respiration after 24 h after treating the BSC with glutamate, which reflects glutamate toxicity. However, we observed a drastic increase in routine respiration (Fig. 2*C*). This increase may still be a result of damage and uncoupling of mitochondria, but it may also reflect elevated ATP production. To dissect respiration linked to ATP production, we treated BSC with oligomycin, an inhibitor of ATP synthase, and observed that the increase in respiration is due to elevated ATP synthesis (Fig. 2*D*). The respiration was also increased after uncoupler titration (Fig. 2*E*), suggesting that this respiratory burst is due to elevated electron flow through ETS and not due to activation of ATP synthase. Next, we tried to understand the mechanism underlying elevated respiration in the presence of glutamate.

It is known that glutamate can upregulate OGDHC, which controls the electron flow from glutamate to the ETS. Alternatively, OGDHC activity can be activated if the levels of NO, a posttranslational regulator of OGDHC, are decreased. To test this hypothesis, we measured OGDHC activity and NO release in our experimental groups. However, as shown in Figure 3, there were no significant changes in OGDHC activity or NO levels, except for a notable increase in OGDHC activity in the glutamate group compared with the memantine group, but not compared with the controls (Fig. 3*A*). These results suggest that the increased mitochondrial respiration observed is not due to modulation of OGDHC activity.

Next, we hypothesized that while TCA cycle activity may be sufficient for ATP synthesis, endogenous glutamate might not be sufficient to fully supply the ETS. To test this, we repeated the experiments, but this time the brain slices were homogenized before assessing mitochondrial function, making the mitochondria accessible to exogenous substrates. We then measured respiration in the presence of excess glutamate (above Km, Michael constant).

The results of this experiment supported our hypothesis (Fig. 4). We measured leak respiration with glutamate (Fig. 4*B*), ATP synthesis-linked respiration with glutamate (Fig. 4*C*), succinate (Fig. 4*D*), and uncoupled respiration (Fig. 4*E*), which in tissue homogenates can be attributed to total ETC capacity. For all these parameters, we did not observe significant differences, though there was a strong trend toward decreased respiration in the glutamate group, suggesting slight mitochondrial damage, which only became apparent when mitochondria were fully loaded with substrate. In the previous experiment, we tested a specific number of brain slices: one slice was enough to measure oxygen flux in Seahorse, and a pool of three was necessary for Oroboros measurements (2 ml chamber).

In the O2k, it was possible to normalize the respiration to tissue weight (Fig. 5). Overall, these results were similar to those obtained from single brain slice, except the respiration in the glutamate group, which tended to be the lowest within all groups, although not significant (Fig. 5). Furthermore, ferrostatin-1, but not memantine, significantly increased the rate of ATP synthesis-linked respiration (Fig. 5*B*) and uncoupled respiration (Fig. 5*D*) compared with the glutamate group. These findings suggest that ferrostatin-1 may have a beneficial effect in our model. This assumption was further supported by the determination of LDH levels, a marker of cell death/damage.

We observed that LDH release was significantly higher in the glutamate groups (Fig. 6*A*,*B*) compared with controls, confirming the toxic effects of glutamate. This increase remained unchanged in the presence of memantine (Fig. 6*A*), suggesting that the cells are not undergoing an excitotoxic pathway. In contrast, treatment with ferrostatin-1 significantly reduced LDH release (Fig. 6*B*), indicating that the toxic effects of glutamate are mediated via the ferroptosis pathway. This aligns with the knowledge that mitochondrial dysfunction is characteristic of the excitotoxic pathway (Nicholls and Budd, 1998; Votyakova and Reynolds, 2005), whereas ferroptosis may operate through damage other than (Wang et al., 2015) mitochondrial cellular membranes, such as the cytoplasmic membrane (Vaglio-Garro et al., 2024b).

Since there is a body of evidence for the activation of the excitotoxic pathway by glutamate, also in our previous publication (Weidinger et al., 2023), we collected more evidence related to the activation of excitotoxic pathway in our model. For this, we repeated several critical experiments with other blockers of glutamate receptors, AP-5 and CNQX, antagonists of NMDA and AMPA glutamate receptors, respectively. The combination of both antagonists covers all glutamate receptors. However, treatment with AP-5 and CNQX, similarly with memantine, did not influence either mitochondrial respiration or the release of LDH (Extended Data Fig. 6-1).

To further confirm the beneficial effects of ferrostatin-1 against glutamate toxicity, we examined cell morphology in brain slices. Neurons are the primary targets of glutamate toxicity, and damaged neurons in the brain tissue typically exhibit eosinophilic cytoplasm and shrunken, pyknotic nuclei. Dead neurons may also exhibit a loss of nuclei. Histological analysis of brain slices (Fig. 7, Table 1) showed changes consistent with the LDH release data. Our data suggest the following scenario for processes occurring in BSC exposed to toxic glutamate concentrations and notably, BSC exhibit several key differences compared with cell cultures. First, the diffusion of glutamate to the cells in BSC is slower than in cell cultures, which means BSC require higher concentrations of glutamate to achieve toxic effects (Humpel, 2015). Second, the tissue structure of BSC may limit the accessibility of certain glutamate receptors, such as those in synaptic clefts that may be blocked by astrocytes. Additionally, some glutamate is taken up by astrocytes before reaching neurons and is converted to glutamine. These factors, slower diffusion, limited receptor accessibility, and astrocyte cooperation, may reduce NMDA receptor activation in BSC compared with cell cultures. As a result, the associated Ca^2+^ influx is slower in BSC than in cell cultures.

Along with Ca^2+^, glutamate enters the cells, whereas as shown in this study, it is used as an energy source to increase ATP synthesis. We propose that the increased ATP production, driven by the influx of glutamate, meets the energy demands required for Ca^2+^ sequestration into mitochondria and the endoplasmic reticulum. This compensatory mechanism may help prevent the development of excitotoxicity.

If tissue slices accumulate glutamate during treatment, intracellular glutamate is higher than in controls. However, homogenate assays use saturating glutamate, eliminating differences in endogenous levels and abolishing the Seahorse effect.

Elevated intracellular Ca^2+^ as consequence of extracellular concentrations of glutamate between 2 and 5 μmol/L leads to excitotoxicity (Mark et al., 2001); ferroptosis is triggered by the extracellular Km of 78 μM glutamate (Lewerenz et al., 2013) which inhibits the Xc-transporter and later inhibiting the intracellular GSH synthesis. It is unlikely that BSC can significantly reduce extracellular glutamate levels and unblock the cystine/glutamate antiporter. This eventually leads to the depletion of intracellular cysteine and reduced GSH, thereby triggering ferroptosis.

Our data provide evidence that glutamate toxicity induces ferroptotic cell death not only in BSC but also in ABSs (Fig. 9). The results indicate that ferroptosis in ABSs is strongly dependent on ACSL4, which is considered the major enzyme responsible for incorporating polyunsaturated fatty acids into membrane phospholipids, thereby providing substrates for lipid hydroperoxides (LOOH) formation. Inhibition of ACSL4 with rosiglitazone significantly reduced both LPO and glutamate-induced cell death (Fig. 8). A similar protective effect was observed with ferrostatin-1, which markedly suppressed LPO and improved cell survival (Fig. 9). Together, these findings strongly support the induction and execution of ferroptosis following glutamate exposure.

Unexpectedly, although deferoxamine almost completely inhibited LPO (Fig. 8), it failed to improve cell survival (Fig. 9). This finding is surprising because deferoxamine is an FDA-approved iron-chelating agent (Yao et al., 2019) and has been reported to inhibit ferroptosis in several experimental disease models (Ma et al., 2016; Bruni et al., 2018).

To explain this discrepancy, we considered several possible mechanisms. First, we considered the activation of an additional iron- and LPO-dependent cell death pathway, such as pyroptosis, which has also been reported to be sensitive to ferrostatin-1. However, this explanation appears unlikely because deferoxamine has been shown to inhibit pyroptosis in several tissues (Zhang et al., 2022; Zhao et al., 2022; Chen et al., 2024), including the brain (Huang et al., 2025). Alternative explanations include the following assumptions:

**1. Deferoxamine may not target the relevant intracellular iron pool.** Neurons contain multiple labile iron pools, and it is possible that the pool critical for ferroptosis initiation is not efficiently accessed by deferoxamine. Recent evidence suggests that the lysosomal iron pool may play a predominant role in ferroptosis (Cañeque et al., 2025). However, in neurons, additional intracellular iron pools may also contribute to ferroptosis induction (Chen et al., 2026).
**2. The pharmacokinetic properties of deferoxamine may limit its neuroprotective efficacy.** We consider this a plausible explanation. Deferoxamine may chelate iron and suppress LPO only after the commitment to cell death has already occurred. In contrast, the longer-lasting actions of ferrostatin-1 and rosiglitazone may allow sustained inhibition of the ferroptotic cascade before irreversible injury develops.
**3. Deferoxamine may exert additional effects on cellular metabolism through inhibition of metalloenzymes.** By depleting intracellular iron, deferoxamine is known to inhibit iron-dependent enzymes such as ribonucleotide reductase and prolyl hydroxylases. Although deferoxamine has much lower affinity for other biologically relevant metal ions than for ferric iron, indirect effects on the function of certain zinc- or copper-dependent enzymes cannot be completely excluded. If deferoxamine impairs antioxidant or prosurvival pathways through inhibition of essential metalloenzymes, these effects could partially offset its antiferroptotic activity resulting from iron chelation.

We hypothesize that in BSC the major mechanism underlying the shift from excitotoxicity to ferroptosis occurs when neurons produce sufficient ATP to handle Ca^2+^ influx via NMDA receptors but are unable to reduce extracellular glutamate levels. While increased ATP production helps prevent excitotoxicity, the inability to lower extracellular glutamate levels ultimately leads to ferroptosis. These findings suggest that in BSC, glutamate toxicity activates distinct pathological pathways compared with dissociated neuronal cultures. This was further supported by our experiments with ABSs, which showed that also in the adult brain glutamate induces ferroptosis. Our data suggest that the ACSL4 is the predominant source of lipids undergoing LPO and inducing ferroptosis. Further research is required to determine which of these pathways predominates in specific pathological conditions associated with neuronal disorders.

## Synthesis

Reviewing Editor: Harald Sontheimer, University of Virginia Decisions are customarily a result of the Reviewing Editor and the peer reviewers coming together and discussing their recommendations until a consensus is reached. When revisions are invited, a fact-based synthesis statement explaining their decision and outlining what is needed to prepare a revision will be listed below. The following reviewer(s) agreed to reveal their identity: Dinesh Verma, Maria Luisa Valle. Note: If this manuscript was transferred from JNeurosci and a decision was made to accept the manuscript without peer review, a synthesis may not be available.

This study addresses an important and timely question regarding the relationship between glutamate toxicity, mitochondrial metabolism, and mechanisms of neuronal cell death in organotypic brain slice cultures. The work is generally well executed, the methodology is robust, and the data analysis is thorough. The findings suggest that toxic concentrations of glutamate intensify neuronal energy metabolism without rescuing cells from death and may shift the dominant mechanism of cell death from excitotoxicity toward ferroptosis. Overall, the study provides interesting and potentially novel insights into glutamate-induced neurodegeneration.

However, several issues need to be addressed to strengthen the central conclusions, improve clarity, and ensure that the claims, particularly regarding ferroptosis, are adequately supported by direct experimental evidence.

1. While the manuscript presents compelling evidence for glutamate-induced cell death and partial protection by ferroptosis inhibitors, the current data do not yet directly demonstrate activation of the ferroptotic machinery. Given that ferroptosis is central to the title and significance statement, additional experiments are strongly recommended to substantiate this claim, including:

Quantitative assessment of intracellular iron levels (e.g., colorimetric assays or fluorescent iron probes), as iron overload is a defining feature of ferroptosis.

Measurement of Acyl-CoA Synthetase Long-Chain Family Member 4 (ACSL4) levels (e.g., Western blot or qPCR), as ACSL4 is a key driver of lipid peroxidation in ferroptotic cell death.

Since ferrostatin-1 was used, assessing Transferrin Receptor 1 expression would help link the observed protection to altered iron uptake and metabolism.

2. As neurons are the primary targets of glutamate toxicity, the manuscript would benefit from clearer identification of neuronal loss. Cresyl Violet (Nissl) staining is recommended, as it allows robust visualization of neuronal cell bodies and dendrites and would strengthen the conclusion that neurons are the main affected population.

3. Clarification and improvement of Seahorse data interpretation. The relationship between Figures 2B and 2C-E needs to be clarified. It is unclear whether panels C-E are derived from the same measurements shown in B, and why the Ferrostatin + Glutamate group does not follow the trend observed in Figure 2B.

Given the reported significant differences, ECAR data should be included in the main manuscript rather than relegated to supplemental material. The rationale for not testing memantine alone and ferrostatin-1 alone in Seahorse assays should be explained.

4. The statement that ferrostatin-1 is beneficial (line ~203) appears to hinge on a single high data point in the glutamate + ferrostatin-1 group. Without this apparent outlier, the difference may not be significant. This claim should be re-evaluated, and appropriate outlier analysis and statistical justification provided.

5. The manuscript should more clearly explain how organotypic brain slice cultures are generated and justify the choice of slices over homogenates. It should be clarified whether this choice is driven by biological relevance, technical constraints of the Seahorse instrument, or both.

6. As acknowledged in the Discussion, measuring intracellular calcium dynamics would significantly strengthen the mechanistic link between glutamate toxicity, excitotoxic signaling, and downstream cell death pathways.

7. The manuscript would benefit from overall improved clarity and organization. Several sections should be rewritten for readability, and figure presentation should be standardized (consistent labeling, sizing, and formatting).

8. The number of biological and technical replicates should be clearly stated for all figures (e.g., Figure 1D).

9. The sample size (n) differs between groups in several figures. This should be clearly justified, and its potential impact on statistical power discussed.

10. Outlier handling requires clarification: The authors should specify whether the ROUT Q test was applied separately to each experimental group. Given concerns about single influential data points, performing Grubbs' test as a complementary analysis (particularly for Figure 5B and D, Glutamate + Ferrostatin-1 groups) is recommended, with revision of conclusions if necessary.

11. Please provide quantification for Figure 7, rather than a qualitative presentation alone.

12. A Western blot demonstrating comparable basal MDH expression in neuronal and astrocytic mitochondria would help validate interpretations related to mitochondrial metabolism.

## References

[B1] Adamchik Y, Frantseva MV, Weisspapir M, Carlen PL, Perez Velazquez JL (2000) Methods to induce primary and secondary traumatic damage in organotypic hippocampal slice cultures. Brain Res Brain Res Protoc 5:153–158. 10.1016/s1385-299x(00)00007-6 10775835

[B2] Andrew RD, et al. (2022) Questioning glutamate excitotoxicity in acute brain damage: the importance of spreading depolarization. Neurocrit Care 37:11–30. 10.1007/s12028-021-01429-4 PMC9259542 35194729

[B3] Bahr BA (1995) Long-term hippocampal slices: a model system for investigating synaptic mechanisms and pathologic processes. J Neurosci Res 42:294–305. 10.1002/jnr.490420303 8583497

[B4] Beach RL, Bathgate SL, Cotman CW (1982) Identification of cell types in rat hippocampal slices maintained in organotypic cultures. Dev Brain Res 3:3–20. 10.1016/0165-3806(82)90071-2 7034866

[B5] Bonora M, Patergnani S, Ramaccini D, Morciano G, Pedriali G, Kahsay AE, Bouhamida E, Giorgi C, Wieckowski MR, Pinton P (2020) Physiopathology of the permeability transition pore: molecular mechanisms in human pathology. Biomolecules 10:1–25. 10.3390/biom10070998 PMC7408088 32635556

[B6] Brooks GA (2018) The science and translation of lactate shuttle theory. Cell Metab 27:757–785. 10.1016/j.cmet.2018.03.008 29617642

[B7] Bruni A, et al. (2018) Ferroptosis-Inducing agents compromise in vitro human islet viability and function article. Cell Death Dis 9:1–10. 10.1038/s41419-018-0506-0 PMC5964226 29789532

[B8] Cañeque T, et al. (2025) Activation of lysosomal iron triggers ferroptosis in cancer. Nature 642:492–500. 10.1038/s41586-025-08974-4 PMC12158755 40335696

[B9] Carrillo E, Romero AM, Gonzalez CU, Turcu AL, Chen S-R, Chen H, Pan H-L, Vázquez S, Twomey EC, Jayaraman V (2024) Memantine inhibits calcium-permeable AMPA receptors. bioRxiv 2024.07.02.601784. 10.1101/2024.07.02.601784 PMC12217772 40593487

[B10] Chen K, Tang Y, Lan L, Li M, Lu Z (2024) Autophagy mediated FTH1 degradation activates gasdermin E dependent pyroptosis contributing to diquat induced kidney injury. Food Chem Toxicol 184:1–13. 10.1016/j.fct.2023.114411 38128689

[B11] Chen W, Tian H, Wei R, Chen X, Jia Y (2026) Ferritin in ferroptosis: implications for neurodegenerative diseases (review). Int J Mol Med 57:1–18. 10.3892/ijmm.2026.5790 PMC12987564 41823531

[B12] Costa I, Barbosa DJ, Benfeito S, Silva V, Chavarria D, Borges F, Remião F, Silva R (2023) Molecular mechanisms of ferroptosis and their involvement in brain diseases. Pharmacol Ther 244:1–32. 10.1016/j.pharmthera.2023.108373 36894028

[B13] Croft CL, et al. (2019) RAAV-Based brain slice culture models of Alzheimer’s and Parkinson’s disease inclusion pathologies. J Exp Med 216:539–555. 10.1084/jem.20182184 PMC6400529 30770411

[B14] Ding K, Liu C, Li L, Yang M, Jiang N, Luo S, Sun L (2023) Acyl-CoA synthase ACSL4: an essential target in ferroptosis and fatty acid metabolism. Chin Med J 136:2521–2537. 10.1097/CM9.0000000000002533 PMC10617883 37442770

[B15] Dixon SJ, et al. (2012) Ferroptosis: an iron-dependent form of nonapoptotic cell death. Cell 149:1060–1072. 10.1016/j.cell.2012.03.042 PMC3367386 22632970

[B16] Doble A (1999) The role of excitotoxicity in neurodegenerative disease: implications for therapy. Pharmacol Ther 81:163–221. 10.1016/S0163-7258(98)00042-4 10334661

[B17] Dobolyi A, Bago A, Palkovits M, Nemeria NS, Jordan F, Doczi J, Ambrus A, Adam-Vizi V, Chinopoulos C (2020) Exclusive neuronal detection of KGDHC-specific subunits in the adult human brain cortex despite pancellular protein lysine succinylation. Brain Struct Funct 225:639–667. 10.1007/s00429-020-02026-5 PMC7046601 31982949

[B18] Donneys A, Ahsan S, Nelson NS, Buchman SR (2013) Reply: deferoxamine restores callus size, mineralization, and mechanical strength in fracture healing after radiotherapy. Plast Reconstr Surg 132:1–2. 10.1097/PRS.0b013e3182a9806a PMC4164207 24281626

[B19] Eguchi K, Velicky P, Hollergschwandtner E, Itakura M, Fukazawa Y, Danzl JG, Shigemoto R (2020) Advantages of acute brain slices prepared at physiological temperature in the characterization of synaptic functions. Front Cell Neurosci 14:1–17. 10.3389/fncel.2020.00063 PMC7096554 32265664

[B20] Elnageh KM, Gaitonde MK (1988) Effect of a deficiency of thiamine on brain pyruvate dehydrogenase: enzyme assay by three different methods. J Neurochem 51:1482–1489. 10.1111/j.1471-4159.1988.tb01115.x 3139833

[B21] Erkartal HŞ, Toy S, Secgin S, Secgin Y (2025) Histopathological and biochemical evaluation of the effects of monosodium glutamate on cognitive memory and brain functions in rats. Eur Food Res Technol 251:1617–1627. 10.1007/s00217-025-04723-z

[B22] Ferreira RS, et al. (2021) Rutin improves glutamate uptake and inhibits glutamate excitotoxicity in rat brain slices. Mol Biol Rep 48:1475–1483. 10.1007/s11033-021-06145-y 33492574

[B23] Friedmann Angeli JP, et al. (2014) Inactivation of the ferroptosis regulator Gpx4 triggers acute renal failure in mice. Nat Cell Biol 16:1180–1191. 10.1038/ncb3064 PMC4894846 25402683

[B24] Gähwiler BH, et al. (1997) Organotypic slice cultures: a technique has come of age. TINS 20:471–477. 10.1016/s0166-2236(97)01122-3 9347615

[B25] García O, Almeida A, Massieu L, Bolaños JP (2005) Increased mitochondrial respiration maintains the mitochondrial membrane potential and promotes survival of cerebellar neurons in an endogenous model of glutamate receptor activation. J Neurochem 92:183–190. 10.1111/j.1471-4159.2004.02851.x 15606907

[B26] Gornall AG, Bardawill CJ, David MM (1949) Determination of serum proteins by means of the biuret reaction. J Biol Chem 177:751–766. 10.1016/S0021-9258(18)57021-6 18110453

[B27] Hotka M, Cagalinec M, Hilber K, Hool L, Boehm S, Kubista H (2020) L-Type Ca2+ channel–mediated Ca2+ influx adjusts neuronal mitochondrial function to physiological and pathophysiological conditions. Sci Signal 13:1–34. 10.1126/scisignal.aaw6923 PMC7116774 32047116

[B28] Hou Y, et al. (2014) Smart soup, a traditional Chinese medicine formula, ameliorates amyloid pathology and related cognitive deficits. PLoS One 9:1–13. 10.1371/journal.pone.0111215 PMC4227681 25386946

[B29] Huang Y, Yang J, Lu T, Shao C, Wan H (2025) Puerarin alleviates cerebral ischemia–reperfusion injury by inhibiting ferroptosis through SLC7A11/GPX4/ACSL4 axis and alleviate pyroptosis through caspase-1/GSDMD axis. Mol Neurobiol 62:8931–8948. 10.1007/s12035-025-04798-5 40056342

[B30] Humpel C (2015) Neuroscience forefront review organotypic brain slice cultures: a review. Neuroscience 305:86–98. 10.1016/j.neuroscience.2015.07.086 PMC4699268 26254240

[B31] Humpel C (2018) Organotypic brain slice cultures. Curr Protoc Immunol 123:1–17. 10.1002/cpim.59 30311739

[B32] Ito S, Yeh FC, Hiolski E, Rydygier P, Gunning DE, Hottowy P, Timme N, Litke AM, Beggs JM (2014) Large-Scale, high-resolution multielectrode-array recording depicts functional network differences of cortical and hippocampal cultures. PLoS One 9:1–16. 10.1371/journal.pone.0105324 PMC4134292 25126851

[B33] Jellinger KA (1999) The role of iron in neurodegeneration prospects for pharmacotherapy of Parkinson’s disease. Drugs Aging 14:115–140. 10.2165/00002512-199914020-00004 10084365

[B34] Kang JB, Park DJ, Son HK, Koh PO (2020) Decrease of protein phosphatase 2A subunit B by glutamate exposure in the cerebral cortex of neonatal rats. Lab Anim Res 36:1–9. 10.1186/s42826-020-00064-y PMC7501672 32995332

[B35] Kokas M, et al. (2025) Microgliosis, neuronal death, minor behavioral abnormalities and reduced endurance performance in alpha-ketoglutarate dehydrogenase complex deficient mice. Redox Biol 85:1–19. 10.1016/j.redox.2025.103743 PMC12271819 40609475

[B36] Komlódi T, Geibl FF, Sassani M, Ambrus A, Tretter L (2018) Membrane potential and delta PH dependency of reverse electron transport-associated hydrogen peroxide production in brain and heart mitochondria. J Bioenerg Biomembr 50:355–365. 10.1007/s10863-018-9766-8 PMC6209044 30116920

[B37] Környei Z, Szlávik V, Szabó B, Gócza E, Czirók A, Madarász E (2005) Humoral and contact interactions in astroglia/stem cell co-cultures in the course of glia-induced neurogenesis. Glia 49:430–444. 10.1002/glia.20123 15546152

[B38] Környei Z, Gócza E, Rühl R, Orsolits B, Vörös E, Szabó B, Vágovits B, Madarász E (2007) Astroglia-derived retinoic acid is a key factor in glia-induced neurogenesis. FASEB J 21:2496–2509. 10.1096/fj.06-7756com 17438145

[B39] Lemasters JJ, Qian T, He L, Kim J-S, Emore SP, Cascio WE, Brenner DA (2002) Role of mitochondrial inner membrane permeabilization in necrotic cell death, apoptosis, and autophagy. Antioxidant Redox Signal 4:769–781. 10.1089/152308602760598918 12470504

[B40] Lewerenz J, et al. (2013) The cystine/glutamate antiporter system Xc- in health and disease: from molecular mechanisms to novel therapeutic opportunities. Antioxidant Redox Signal 18:522–555. 10.1089/ars.2011.4391 PMC3545354 22667998

[B41] Li LB, Chai R, Zhang S, Xu SF, Zhang YH, Li HL, Fan YG, Guo C (2019) Iron exposure and the cellular mechanisms linked to neuron degeneration in adult mice. Cells 8:1–17. 10.3390/cells8020198 PMC6406573 30813496

[B42] Li Q, et al. (2017) Inhibition of neuronal ferroptosis protects hemorrhagic brain. JCI Insight 2:1–19. 10.1172/jci.insight.90777 PMC5374066 28405617

[B43] Ma S, Henson ES, Chen Y, Gibson SB (2016) Ferroptosis is induced following siramesine and lapatinib treatment of breast cancer cells. Cell Death Dis 7:802–809. 10.1038/cddis.2016.208 PMC4973350 27441659

[B44] Ma S, et al. (2023) Sustained antidepressant effect of ketamine through NMDAR trapping in the LHb. Nature 622:802–809. 10.1038/s41586-023-06624-1 PMC10600008 37853123

[B45] Mark LP, Prost RW, Ulmer JL, Smith MM, Daniels DL, Strottmann JM, Brown WD, Hacein-Bey L (2001) Pictorial review of glutamate excitotoxicity: fundamental concepts for neuroimaging. AJNR Am J Neuroradiol 22:1813–1824. PMC7973850 11733308

[B46] McKenna MC, Stridh MH, McNair LF, Sonnewald U, Waagepetersen HS, Schousboe A (2016) Glutamate oxidation in astrocytes: roles of glutamate dehydrogenase and aminotransferases. J Neurosci Res 94:1561–1571. 10.1002/jnr.23908 27629247

[B47] Mkrtchyan GV, Graf A, Trofimova L, Ksenofontov A, Baratova L, Bunik V (2018) Positive correlation between rat brain glutamate concentrations and mitochondrial 2-oxoglutarate dehydrogenase activity. Anal Biochem 552:100–109. 10.1016/j.ab.2018.01.003 29326069

[B48] Nicholls DG, Budd SL (1998) Mitochondria and neuronal glutamate excitotoxicity. Biochim Biophys Acta 1366:97–112. 10.1016/S0005-2728(98)00123-6 9714760

[B49] Ortiz-Rodriguez JM, Bucci D, Tovar-Pascual L, Granata S, Spinaci M, Nesci S (2024) Analysis of stallion spermatozoa metabolism using agilent seahorse XFp technology. Anim Reprod Sci 271:1–8. 10.1016/j.anireprosci.2024.107633 39509949

[B50] Ray SK, Dixon CE, Banik NL (2002) Molecular mechanisms in the pathogenesis of traumatic brain injury. Histol Histopathol 17:1137–1152. 10.14670/HH-17.1137 12371142

[B51] Reynolds IJ, Hastings TG (1995) Glutamate induces the production of reactive oxygen species in cultured forebrain neurons following NMDA receptor activation. J Neurosci 15:3318–3327. 10.1523/JNEUROSCI.15-05-03318.1995 PMC6578215 7751912

[B52] Robert F, Bert L, Stoppini L (2002) Blockade of NMDA-receptors or calcium-channels attenuates the ischaemia-evoked efflux of glutamate and phosphoethanolamine and depression of neuronal activity in rat organotypic hippocampal slice cultures. C R Biol 325:495–504. 10.1016/s1631-0691(02)01451-8 12161929

[B53] Sebastião AM, De Mendonça A, Moreira T, Ribeiro JA (2001) Activation of synaptic NMDA receptors by action potential-dependent release of transmitter during hypoxia impairs recovery of synaptic transmission on reoxygenation. J Neurosci 21:8564–8571. 10.1523/JNEUROSCI.21-21-08564.2001 PMC6762820 11606644

[B54] Sieg F, Obst K, Gorba T, Riederer B, Pape H-C, Wahle P (1998) Postnatal expression pattern of calcium-binding proteins in organotypic thalamic cultures and in the dorsal thalamus in vivo. Develop Brain Res 110:83–95. 10.1016/S0165-3806(98)00097-2 9733927

[B55] Simoni A, Griesinger CB, Edwards FA (2003) Development of rat CA1 neurones in acute versus organotypic slices: role of experience in synaptic morphology and activity. J Physiol 550:135–147. 10.1113/jphysiol.2003.039099 PMC2343027 12879864

[B56] Smirnova YD, et al. (2025) Osteosarcoma cells and undifferentiated human mesenchymal stromal cells are more susceptible to ferroptosis than differentiated human mesenchymal stromal cells. Antioxidants 14:1–6. 10.3390/antiox14020189 PMC11852062 40002376

[B57] Sonnay S, Gruetter R, Duarte JMN (2017) How energy metabolism supports cerebral function: insights from 13C magnetic resonance studies in vivo. Front Neurosci 11:1–20. 10.3389/fnins.2017.00288 PMC5445183 28603480

[B58] Sonnewald U, Schousboe A (2016) Introduction to the glutamate–glutamine cycle. Adv Neurobiol 13:1–7. 10.1007/978-3-319-45096-4_1 27885624

[B59] Staal JA, Alexander SR, Liu Y, Dickson TD, Vickers JC (2011) Characterization of cortical neuronal and glial alterations during culture of organotypic whole brain slices from neonatal and mature mice. PLoS One 6:1–10. 10.1371/journal.pone.0022040 PMC3137607 21789209

[B60] Stoppini L, Buchs P-A, Muller D (1991) A simple method for organotypic cultures of nervous tissue. J Neurosci Methods 37:173–182. 10.1016/0165-0270(91)90128-M 1715499

[B61] Stoyanovsky DA, et al. (2019) Iron catalysis of lipid peroxidation in ferroptosis: regulated enzymatic or random free radical reaction? Free Radical Biol Med 133:153–161. 10.1016/j.freeradbiomed.2018.09.008 PMC6555767 30217775

[B62] Sváb G, Kokas M, Sipos I, Ambrus A, Tretter L (2021) Methylene blue bridges the inhibition and produces unusual respiratory changes in Complex Iii-inhibited mitochondria. Studies on rats, mice and Guinea pigs. Antioxidants 10:1–22. 10.3390/antiox10020305 PMC7920423 33669457

[B63] Szabó I, Zoratti M (1992) The mitochondrial megachannel is the permeability transition pore I. J Bioenerg Biomembr 24:111–117. 10.1007/BF00769537 1380498

[B64] Tang Y-G, Zucker RS (1997) Mitochondrial involvement in post-tetanic potentiation of synaptic transmission. Neuron 18:483–491. 10.1016/s0896-6273(00)81248-9 9115741

[B65] Tretter L, Adam-Vizi V (2000) Inhibition of Krebs cycle enzymes by hydrogen peroxide: a key role of-ketoglutarate dehydrogenase in limiting NADH production under oxidative stress. J Neurosci 20:8972–8979. 10.1523/JNEUROSCI.20-24-08972.2000 PMC6773008 11124972

[B66] Underwood E, Redell JB, Zhao J, Moore AN, Dash PK (2020) A method for assessing tissue respiration in anatomically defined brain regions. Sci Rep 10:1–14. 10.1038/s41598-020-69867-2 PMC7413397 32764697

[B67] Vaglio-Garro A, Halasz A, Nováková E, Gasser AS, Zavadskis S, Weidinger A, Kozlov AV (2024a) Interplay between energy supply and glutamate toxicity in the primary cortical culture. Biomolecules 14:1–19. 10.3390/biom14050543 PMC11118065 38785950

[B68] Vaglio-Garro A, Kozlov AV, Smirnova YD, Weidinger A (2024b) Pathological interplay between inflammation and mitochondria aggravates glutamate toxicity. Int J Mol Sci 25. 10.3390/ijms25042276 PMC10889519 38396952

[B69] Verstreken P, Ly CV, Venken KJT, Koh TW, Zhou Y, Bellen HJ (2005) Synaptic mitochondria are critical for mobilization of reserve pool vesicles at *Drosophila* neuromuscular junctions. Neuron 47:365–378. 10.1016/j.neuron.2005.06.018 16055061

[B70] Votyakova TV, Reynolds IJ (2005) Ca2+-induced permeabilization promotes free radical release from Rat brain mitochondria with partially inhibited Complex I. J Neurochem 93:526–537. 10.1111/j.1471-4159.2005.03042.x 15836612

[B71] Wang T, et al. (2022a) Ferroptosis mediates selective motor neuron death in amyotrophic lateral sclerosis. Cell Death Differ 29:1187–1198. 10.1038/s41418-021-00910-z PMC9177596 34857917

[B72] Wang W, Zhang F, Li L, Tang F, Siedlak SL, Fujioka H, Liu Y, Su B, Pi Y, Wang X (2015) MFN2 couples glutamate excitotoxicity and mitochondrial dysfunction in motor neurons. J Biol Chem 290:168–182. 10.1074/jbc.M114.617167 PMC4281719 25416777

[B73] Wang ZL, Yuan L, Li W, Li JY (2022b) Ferroptosis in Parkinson’s disease: glia–neuron crosstalk. Trends Mol Med 28:258–269. 10.1016/j.molmed.2022.02.003 35260343

[B74] Weidinger A, Dungel P, Perlinger M, Singer K, Ghebes C, Duvigneau JC, Müllebner A, Schäfer U, Redl H, Kozlov AV (2013) Experimental data suggesting that inflammation mediated rat liver mitochondrial dysfunction results from secondary hypoxia rather than from direct effects of inflammatory mediators. Front Physiol 4:1–9. 10.3389/fphys.2013.00138 PMC3675332 23760194

[B75] Weidinger A, et al. (2023) Oxoglutarate dehydrogenase complex controls glutamate-mediated neuronal death. Redox Biol 62:1–15. 10.1016/j.redox.2023.102669 PMC10031542 36933393

[B76] Wong SB, Cheng SJ, Hung WC, Lee WT, Min MY (2015) Rosiglitazone suppresses in vitro seizures in hippocampal slice by inhibiting presynaptic glutamate release in a model of temporal lobe epilepsy. PLoS One 10:1–14. 10.1371/journal.pone.0144806 PMC4685987 26659605

[B77] Yang WS, Stockwell BR (2008) Synthetic lethal screening identifies compounds activating iron-dependent, nonapoptotic cell death in oncogenic-RAS-harboring cancer cells. Chem Biol 15:234–245. 10.1016/j.chembiol.2008.02.010 PMC2683762 18355723

[B78] Yao X, et al. (2019) Deferoxamine promotes recovery of traumatic spinal cord injury by inhibiting ferroptosis. Neural Regen Res 14:532–541. 10.4103/1673-5374.245480 PMC6334606 30539824

[B79] Youdim MBH, Grünblatt E, Levites Y, Maor GG, Mandel S (2002) Early and late molecular events in neurodegeneration and neuroprotection in Parkinson’s disease MPTP model as assessed by CDNA microarray; the role of iron. Neurotox Res 4:679–689. 10.1080/1029842021000045507 12709306

[B80] Zhang F, Liu T, Huang HC, Zhao YY, He M, Yuan W, Li L, Li J, Wu DM, Xu Y (2022) Activation of pyroptosis and ferroptosis is involved in radiation-induced intestinal injury in mice. Biochem Biophys Res Commun 631:102–109. 10.1016/j.bbrc.2022.09.073 36183550

[B81] Zhao S, Ge C, Li Y, Chang L, Dan Z, Tu Y, Deng L, Kang H, Li C (2022) Desferrioxamine alleviates UHMWPE particle-induced osteoclastic osteolysis by inhibiting caspase-1-dependent pyroptosis in osteocytes. J Biol Eng 16:1–18. 10.1186/s13036-022-00314-8 PMC9733322 36482442

[B82] Zhong Y, Gao Y, Zhou D, Ma X, Chen H, Xu Y, Yang W, Yu X (2022) Structural basis for the activity and regulation of human α-ketoglutarate dehydrogenase revealed by cryo-EM. Biochem Biophys Res Commun 602:120–126. 10.1016/j.bbrc.2022.02.093 35272141

[B83] Zou JY, Crews FT (2005) TNFα potentiates glutamate neurotoxicity by inhibiting glutamate uptake in organotypic brain slice cultures: neuroprotection by NFκB inhibition. Brain Res 1034:11–24. 10.1016/j.brainres.2004.11.014 15713255

